# Worth Less?: Why Men (and Women) Devalue Care-Oriented Careers

**DOI:** 10.3389/fpsyg.2018.01353

**Published:** 2018-08-10

**Authors:** Katharina Block, Alyssa Croft, Toni Schmader

**Affiliations:** ^1^Department of Psychology, The University of British Columbia, Vancouver, BC, Canada; ^2^Department of Psychology, The University of Arizona, Tucson, AZ, United States

**Keywords:** gender differences, agentic values, communal values, career evaluations, career choice, career status, occupational interest

## Abstract

In the present research, we applied a goal-congruity perspective – the proposition that men and women seek out roles that afford their internalized values (Diekman et al., 2017) – to better understand the degree to which careers in healthcare, early education, and domestic roles (HEED; Croft et al., 2015) are devalued in society. Our first goal was to test the hypothesis that men, relative to women, are less interested in pursuing HEED careers in part because they are less likely than women to endorse communal values. A second, more novel goal was to extend goal congruity theory to examine whether gender differences in communal values also predict the belief that HEED careers add worth to society and are deserving of higher salaries. In three studies of undergraduate students (total *N* = 979), we tested the predictive role of communal values (i.e., a focus on caring for others), as distinct from agentic values (i.e., a focus on status, competition, and wealth; Bakan, 1966). Consistent with goal congruity theory, Studies 1 and 2 revealed that men’s lower interest in adopting HEED careers, such as nursing and elementary education, was partially mediated by men’s (compared to women’s) lower communal values. Extending the theory, all three studies also documented a general tendency to see HEED as having relatively lower worth to society compared to STEM careers. As expected, communal values predicted perceiving higher societal worth in HEED careers, as well as supporting increases in HEED salaries. Thus, gender differences in communal values accounted for men’s (compared to women’s) tendency to perceive HEED careers as having less societal worth and less deserving of salary increases. In turn, gender differences in perceived societal worth of HEED itself predicted men’s relatively lower interest in pursuing HEED careers. In no instance, did agentic values better explain the gender difference in HEED interest or perceived worth. These findings have important implications for how we understand the value that society places on occupations typically occupied by women versus men.

## Introduction

“If we’re going to get to real equality between men and women, we have to focus less on women and more on elevating the value of care.”

- Anne-Marie Slaughter

Try for a moment, to imagine a world without teachers and nurses. Not only is this difficult to do, but it also paints an unpleasant picture. Workers in healthcare and education play vital roles in the functioning of civil societies (Bordieu and Passeron, 1990; Holmes and Gastaldo, 2002). Yet, as political scientist and policy analyst [Slaughter (2015), October 1] suggests, these positions are often devalued. On the one hand, men devalue care-oriented occupations (e.g., teaching or nursing) as personal career paths (Croft et al., 2015). But in addition, those men and women who do choose healthcare, early education, and domestic roles (HEED; Croft et al., 2015) are afforded both lower status and lower salaries in many societies (Cross and Bagilhole, 2002; England et al., 2002). Many HEED professionals feel this broader devaluation. In the United States, public funding for education has been cut by as much as 37% since 2008 – prompting teacher-strikes in several states to protest low salaries (Turner, 2018, April 11). Given the important role of these care-oriented professions to personal (Le et al., 2018) and societal well-being (Bordieu and Passeron, 1990; Holmes and Gastaldo, 2002), why are HEED careers not highly valued, both as occupational choices for men and for society as a whole? In the current research, we apply a goal congruity perspective (Diekman et al., 2017) to test whether men’s and women’s endorsement of communal values predicts their personal interest in and perceptions of the broader societal worth of HEED careers.

### Gendered Career Perceptions

Despite several waves of feminism and active efforts by governments, men and women continue to be disproportionately represented in different types of occupations. To date, women remain underrepresented in science, technology, engineering, and math (STEM) fields, where they make up only 9–16% of engineers and 21% of computer programmers (Bureau of Labor Statistics, 2017). Whereas an active literature seeks to understand and rectify this underrepresentation of women in STEM, much less attention has been paid to the equally sizable gender imbalance in communally oriented careers (see Croft et al., 2015). In many HEED careers, men are markedly underrepresented, making up only 10% of nurses and 4% of preschool and kindergarten teachers in the United States (Bureau of Labor Statistics, 2017). Men’s self-selection out of care-oriented roles might have negative consequences both for men themselves and those served by HEED professionals (Croft et al., 2015). Thus, the first goal of the current research was to better understand why men are relatively less interested in personally pursuing HEED careers.

As suggested by Slaughter, HEED occupations are not simply unpopular career choices among men, they are also generally devalued in society. HEED careers are assigned lower status and paid lower salaries than traditionally male-dominated STEM careers (e.g., England et al., 2001). In the United States, where teachers stage walk-outs to protest their low salaries, teaching is among the lowest paid occupations given training requirements (Alegretto and Mishel, 2016). Similarly, in other Western countries such as the United Kingdom and Germany, hourly pay-rates in education and healthcare are considerably lower than those in scientific sectors (ILOSTAT, 2015). Such data suggest that HEED careers are, quite literally, perceived as *worth* less money than are STEM careers. And because women tend to be overrepresented in these low-paying HEED careers, sociologists have suggested that the tendency to undervalue care-oriented roles perpetuates the persistent gender wage gap women continue to face in modern societies (Kilbourne et al., 1994; England et al., 2001). Despite such broad implications for important social issues, to the best of our knowledge, there has been no empirical social-psychological investigation of the perceived societal worth of HEED (or STEM) careers. Thus, in addition to better understanding men’s disinterest in HEED careers, our second and perhaps more important aim was to document whether people do in fact see HEED careers as having less worth than STEM careers, and if so, identify factors that predict this perception. We examine these questions through the lens of social role theory, goal congruity theory, and status-value theory.

### Social Role Theory and Goal Congruity

Social role theory (Eagly, 1987; Eagly and Wood, 2012) provides a broad framework for understanding how gender segregation into different roles eventually leads new generations of men and women to internalize distinct traits and values. The theory suggests that the historical overrepresentation of women in care-oriented (e.g., HEED) roles results in societal gender stereotypes of women as inherently more communal (i.e., oriented toward care for others, Bakan, 1966) than men. In turn, such stereotypical expectations lead new generations of women to internalize communal values more than do men (Eagly, 1987; Ridgeway and Correll, 2004; Abele, 2003; Eagly and Wood, 2012). In line with this theory, a wealth of evidence shows that men endorse communal values and traits relatively less than do women (Bem, 1974; Spence et al., 1974; Spence and Helmreich, 1978; Costa et al., 2001; Donnelly and Twenge, 2017). Moreover, such gender differences in communal values are evident early in development, with boys reporting lower communal value endorsement than girls as early as age 6 (Block et al., 2018). In contrast, although women are viewed as less agentic (i.e., focused on self-promotion, Bakan, 1966) than men (Bem, 1974; Spence et al., 1974; Spence and Helmreich, 1978), women have become somewhat more agentic as they have entered the workforce (Donnelly and Twenge, 2017).

Once men and women have internalized communal (and other) values to different extents, these values should, in turn, color their perceptions of careers. As an extension of social role theory, the goal congruity perspective (Diekman et al., 2017) suggests that both men and women seek careers that match their own internalized values for communion and agency. Female-stereotypic (e.g., HEED) and male-stereotypic (e.g., STEM) careers differ in the extent to which they are perceived to afford these values. Specifically, HEED-related careers, such as nursing, are perceived as highly communal but lower in agency; whereas STEM careers are perceived as relatively lower in communion but higher in agency (Diekman et al., 2010; Tellhed et al., 2018). As a consequence, the goal congruity perspective offers an explanation for patterns of horizontal gender segregation by occupation. Past findings show that women’s relatively higher communal value endorsement predicts a reduced interest in taking on STEM and other male-dominated careers (Evans and Diekman, 2009; Diekman et al., 2010; Diekman and Steinberg, 2013). In addition, reframing STEM careers as more communal increases women’s interest in these careers (Diekman et al., 2011). Though not exclusively focused on HEED careers, past research also suggests that endorsing communal goals predicts favoring female-stereotypic careers among undergraduate (Evans and Diekman, 2009) and high school students (Tellhed et al., 2018). Thus, the first goal of the present research was to test the straightforward prediction that men’s lower interest in HEED careers is partly explained by their lower endorsement of communal values.

The current research also extends the goal congruity perspective beyond merely understanding men’s and women’s career choices, to examine the broader worth that men (and women) perceive in HEED and STEM fields. In line with the introductory quote by Anne-Marie Slaughter, we begin by hypothesizing that, although both men and women might see HEED careers as having less worth compared to STEM, men might particularly devalue the importance of HEED to society. Previous work on status-value asymmetries suggests that high-status group members tend to devalue domains in which their group is underrepresented, whereas low-status group members find it difficult to devalue domains inhabited by higher-status outgroups (Schmader et al., 2001). Given men’s higher status in society (Conway et al., 1996; Correll, 2004; Ridgeway and Correll, 2004), we expect them to see less value in female-dominated HEED careers than do women, whereas women might not similarly devalue the broader societal worth of male-dominated STEM careers.

In addition to the importance granted to the roles occupied by higher-status groups, we propose that goal congruity processes also shape the perceived worth of various careers. Specifically, we theorized that internalized values not only guide men’s and women’s personal career choices, but also their broader perceptions of careers as adding (or not adding) significant worth to society. Because HEED careers are seen as supporting communal goals (Diekman et al., 2010), we expected those that those who feel that communion is broadly important (who tend to be women) will see greater worth in HEED careers’ contributions to society and will want to see HEED workers compensated well. And because men tend to endorse communal values less strongly, we predicted that men will perceive relatively less societal worth in HEED careers than will women – a difference that will be mediated by men’s lower endorsement of communal values.

An additional, more exploratory goal of these studies was to examine whether men’s less-favorable perceptions of the societal worth of HEED careers would subsequently predict their reduced interest in actually pursuing HEED careers. Generally speaking, people seek careers that they perceive as making meaningful contributions to society (Hirschi, 2012). What is seen as meaningful, however, could vary based on one’s personal values. Thus, we also explored whether men’s tendency to perceive relatively less worth in HEED roles (as predicted by their relatively lower communal value endorsement) would mediate gender differences in interest in HEED careers.

Although our primary focus was on communal value endorsement as a predictor of HEED evaluations, we also examined other values that might be seen as incompatible with HEED. Perhaps men are relatively less interested (or perceive less worth) in HEED careers not because they place less value on communion, but instead because they care more about agency, competition, and or money. These other values might feel incompatible with careers that seem to emphasize putting others’ needs above one’s own and putting others’ well-being above profit. Evidence of gender differences in agency is mixed. Some contemporary studies no longer show gender differences in agentic values (Diekman et al., 2010), but in other research men rate themselves higher on agentic traits than do women (Donnelly and Twenge, 2017). In addition, teenage boys are more likely than girls to prioritize agentic over communal goals (Tellhed et al., 2018), and men are more likely to emphasize competition as a means to gain status (Gneezy and Rustichini, 2004; Croson and Gneezy, 2009) and focus on salary when evaluating careers (Fortin, 2017). If HEED careers are perceived as not affording competition and wealth, these professions could represent a mismatch to men’s values, providing an alternative or independent reason for their devaluation of HEED.

### Overview of Research

In three samples of young adults, we examined the relationship between gender, personal values, and evaluations of HEED careers as both: (a) personally interesting, and (b) as having broader worth to society. In Studies 1 and 2, we applied the goal congruity perspective to men’s HEED interest, and tested the hypothesis that men are less interested in HEED careers to the extent that they hold less communal values than do women. We also hypothesized that HEED careers would be seen as having less worth to society compared to STEM. More importantly, we expected men, as compared to women, to perceive HEED careers as having less societal worth (Studies 1–3) and less deserving of pay increases (Study 3), two effects that would be partly explained by men’s lower communal values. A more exploratory prediction was that men’s relatively low interest in HEED careers as a function of their lower communal values would itself be partially explained by the lower societal worth men grant these occupations (Studies 1 and 2). Whereas we focused on communal values across hypotheses, we also tested the additional explanations that agentic values (Studies 1–3), trait competitiveness (Study 2), and/or material values (Study 3) would predict negative HEED evaluations instead of, or in addition to, communal values. Lastly, we also assessed men’s and women’s interest in, and perceived worth of STEM careers for comparison.

## Study 1

One goal of Study 1 was to extend previous work on goal congruity theory (Diekman et al., 2017) to formally test the hypothesis that men’s, compared to women’s, relatively lower interest in HEED careers can be explained by their lower communal values. The second and more novel goal was to examine whether communal values also predict perceptions of the broader societal worth of HEED careers. Finally, we tested whether there is a gender difference in the perceived societal worth of HEED careers that might be mediated by the gender gap in communal (and/or agentic) values.

### Method

#### Participants and Procedure

Trained research assistants (male and female) recruited 380 (184 male/196 female) participants in public areas of a large Canadian university to complete a brief paper-and-pencil survey about “values, opinions, and preferences” in exchange for candy. Although no *a priori* power analysis was conducted, a sensitivity analysis suggested that the study was powered to detect a small to medium sized interaction in an analysis of variance ($\eta_{p}^{2}$ = 0.02) at 80% power. Participants had a mean age of 19.91 years (*SD* = 2.02) and were predominantly East Asian (46.3%) and Caucasian (22.9%), with some South East Asian (13.4%) participants.^1^

#### Measures

##### Personal values

Participants rated “how important” each of seven communal values (helping others, serving humanity, working with people, connection with others, attending to others, caring for others, intimacy; α = 0.79) and seven agentic values (power, recognition, achievement, self-promotion, independence, status, competition; α = 0.69) were “to them personally.” This list of values was adapted from Diekman et al. (2010). Participants rated each value by placing an *X* on a 10 cm long scale anchored by “Not at all important” (0) to “Extremely important” (100). Responses were measured with a millimeter ruler.

##### Career interest

To assess personal interest, participants rated the “degree to which [they] can imagine being at all interested in” five HEED (social worker, human resources manager, preschool/kindergarten teacher, educational administrator, registered nurse; α = 0.76) and five STEM careers (engineer, computer scientist, environmental scientist, architect, dentist; α = 0.68) adapted from Diekman et al. (2010).^2^ Ratings were made on the same 10 cm response-scale used for values, with the anchors “Not at all interested” to “Extremely interested”.

##### Perceived worth to society

Participants rated the same HEED and STEM careers (α_HEED_ = 0.90; α_STEM_ = 0.85) for their perceived worth to society. Specifically, participants estimated the *ideal pay* they would assign to reflect a given career’s worth to society. We emphasized that “we are NOT asking [them] to estimate the *actual* pay these roles currently get on the job market, but rather the VALUE you want to assign to them.” Ratings were made by placing an *X* on a visual continuous scale with the anchors of “$0 per hour” to “$400 per hour”.^3^

##### Exploratory variables and demographics

The survey included exploratory measures of future breadwinner and caregiver roles and career- vs. family prioritization. Gender differences in these “domestic” variables, and their relationships to personal values can be found in the **Supplementary Online Materials** (SOM) but will not be discussed in this paper. At the end of the study, participants completed a standard demographic questionnaire including gender, age, year standing, major, ethnicity, sexual orientation and dating status.

The full datasets all studies in this manuscript can be located at osf.io/ejz78.

### Results

#### Gender Differences

##### Personal values

Based on previous findings, we expected men to score lower than women on communal values but expected no clear gender difference in agency (Diekman et al., 2017). In line with this prediction, a 2 (participants gender: male vs. female) × 2 (value-type: communal vs. agentic) mixed analysis of variance (ANOVA) yielded a significant interaction between participant gender and value-type, *F*(1,376) = 11.62, *p* = 0.001, $\eta_{p}^{2}$ = 0.03. Pairwise comparisons revealed that men valued communion less than did women, *p* < 0.001, but men and women valued agency to a similar extent, *p* = 0.970. It is notable, however, that both men and women reported valuing communion more than agency, *all p*s < 0.001. Descriptive statistics, *d*-scores for gender differences, and correlations for key variables are reported in **Table 1**.

**Table 1:** Study 1 descriptive statistics and bivariate correlation.

	1	2	3	4	5	6
(1) Communal values	–					
(2) Agentic values	**0.15^∗^**	–				
(3) HEED interest	**0.27^∗^**	0.04	–			
(4) HEED value	**0.19^∗^**	0.03	**0.29^∗^**	–		
(5) STEM interest	-0.07	0.02	**0.28^∗^**	0.02	–	
(6) STEM value	0.11	0.10	**0.17^∗^**	**0.76^∗^**	0.05	–

*M*_men_ (*SD*)	69.44 (15.38)	57.79 (14.30)	31.28 (19.25)	163.11 (78.34)	41.62 (19.77)	203.91 (77.47)
*M*_Women_ (*SD*)	75.71 (12.68)	57.73 (13.46)	44.74 (19.47)	187.23 (81.36)	35.46 (20.24)	216.55 (72.12)
*d-score_(men-women)_*	**-0.44^∗^**	0.004	**-0.68^∗^**	**-0.30^∗^**	**0.31^∗^**	**-**0.17

*^∗^p < 0.05. Superscripts on d-scores indicate significant level of gender differences from tests reported in text. All scales had a range of 0–100 except the value measures, in which participants expressed value in an hourly pay amount that could vary between $0–$400/hr.*

##### Career interest

Reflecting past evidence of gender differences in occupational interest (Evans and Diekman, 2009; Su et al., 2009; Diekman et al., 2017), we expected men to be more interested in male-stereotypic STEM careers, and women to be more interested in female-stereotypic HEED careers. A 2 (participant gender) × 2 (career-type; STEM vs. HEED) mixed ANOVA yielded the expected gender × career-type interaction, *F*(1,378) = 2.65, *p* < 0.001, $\eta_{p}^{2}$ = 0.16. As expected, men reported significantly less interest in HEED careers than did women, *p* < 0.001. Women, in turn, reported less interest in STEM careers than did men, *p* = 0.003. Pairwise comparisons showed that women favored HEED over STEM careers, *p* < 0.001, whereas men favored STEM over HEED careers, *p* < 0.001.

##### Perceived worth to society

**FIGURE 1:**
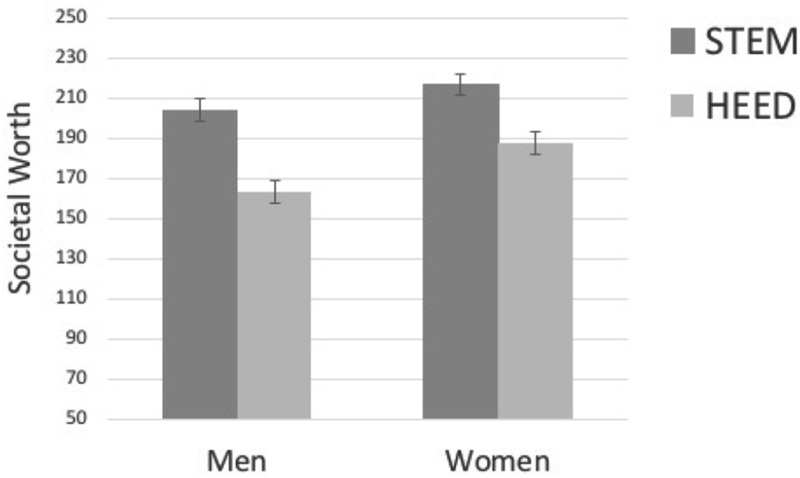
Study 1 Perceived societal worth of HEED and STEM by participant gender. Error bars represent standard errors of the mean.

We expected that men, as compared to women, would see less societal worth in HEED careers (as indicated by a lower ideal salary). A 2 (participant gender) × 2 (career-type) mixed ANOVA on the perceived societal worth assigned to these careers revealed significant main effects of career-type, *F*(1,378) = 156.81, *p* < 0.001, $\eta_{p}^{2}$ = 0.29; and of gender, *F*(1,378) = 6.29, *p* = 0.013, $\eta_{p}^{2}$ = 0.02; that were qualified by a significant gender by career-type interaction, *F*(1,378) = 4.78, *p* = 0.029, $\eta_{p}^{2}$ = 0.01. Consistent with key hypotheses, and as shown in **Figure 1**, men assigned significantly less societal worth to HEED careers than did women, *p* = 0.003. However, women and men assigned similarly high levels of societal worth to STEM careers, *p* = 0.100. Although both men and women assigned higher ideal salaries to STEM than to HEED, *p*s < 0.001, that difference was significantly smaller for women, *d* = 0.38, compared to men, *d* = 0.52.

#### Mediation of Occupational Perceptions by Communal Values

**FIGURE 2:**
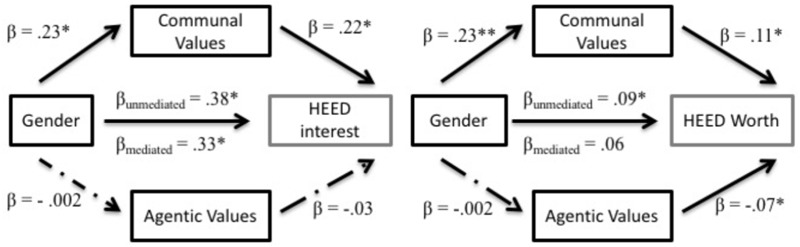
Mediation models for HEED variables in Study 1. All mediators were entered simultaneously for each model. ^∗^*p* < 0.05, ^∗∗^*p* < 0.01.

Given the observed gender differences in career interest and perceived societal worth of HEED careers, we next tested our hypotheses that communal values would partially account for these gender gaps in HEED perceptions. Using Preacher and Hayes’ (2012) PROCESS Macro in SPSS (Hayes, 2012; Model 4), we regressed each outcome variable (personal interest and societal worth of HEED in separate models) onto gender as the predictor variable and communal values and agentic values as simultaneous mediator variables. To focus specifically on the relationship of communal values with HEED perceptions, analyses always controlled parallel ratings of STEM occupations.^4^ All variables were standardized in these and other models. Indirect effects of gender on career via values, and their confidence intervals, were estimated using 10,000 bootstrapped re-samples. Models are visualized in **Figure 2**.

In addition to the already described gender differences in communal values, β = 0.23, *SE* = 0.05, *t*(376) = 4.58, *p* < 0.001; communal values predicted significantly higher personal interest in HEED careers, β = 0.22, *SE* = 0.05, *t*(373) = 4.76, *p* < 0.001, as well as higher societal worth assigned to HEED careers, β = 0.11, *SE* = 0.04, *t*(373) = 3.13, *p* = 0.002. Importantly, there was a significant indirect effect of gender on HEED interest as mediated by communal values, *a^∗^b* = 0.05, *SE* = 0.02, bootstrapped *CI*_0.95_ (0.02, 0.09), *p* < 0.05; and of gender on perceived societal worth of HEED careers as mediated by communal values, *a^∗^b* = 0.02, *SE* = 0.01, bootstrapped *CI*_0.95_ (0.01, 0.05), *p* < 0.05. In contrast, endorsing agentic values did not predict personal interest in HEED, β = -0.003, *SE* = 0.05, *t*(373) = -0.06, *p* = 0.956, but did predict lower perceived societal worth of HEED in this sample, β = -0.07, *SE* = 0.03, *t*(373) = -1.98, *p* = 0.049. Yet, the lack of gender difference in agentic values precludes this variable from mediating effects, both *a^∗^b*s < 0.001.

These mediation models provide support for the hypothesis that men show less personal interest in and assign lower societal worth to HEED careers, in part, because communal values are less important to them than they are to women (13% of total gender difference in career interest and 33% of gender difference societal worth was explained by communal values). After entering communal and agentic values (alongside STEM perceptions as control) into these models, gender was still a significant predictor of HEED interest, β = 0.33, *SE* = 0.05, *t*(373) = 7.10, *p* < 0.001, but not of societal worth of HEED, β = 0.06, *SE* = 0.03, *t*(373) = 1.90, *p* = 0.058.^5^

##### Are gender differences in HEED interest mediated by communal values and societal worth?

Given that gender differences in communal values related to both gender differences in HEED interest and societal worth, we further asked whether men’s, compared to women’s, relatively lower interest in HEED careers is partly explained by the lower perceived worth of these careers. We tested this serial mediation with the PROCESS macro (Hayes, 2012; model 6) entering gender as the main predictor, communal values as mediator 1, and societal worth of HEED as mediator 2 of a model predicting HEED interest as the outcome. All paths controlled for agentic values, societal worth of STEM, and interest in STEM.

**FIGURE 3:**
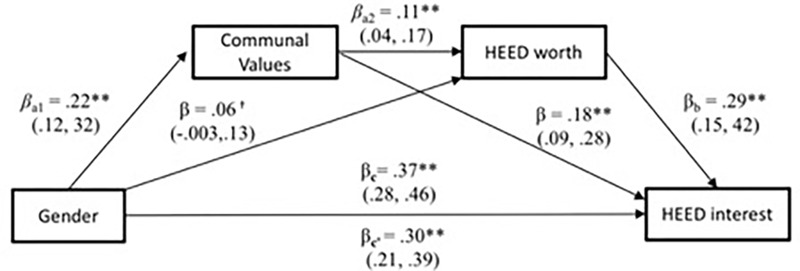
Serial mediation model for HEED variables in Study 1. ^∗∗^*p* < 0.01, ^∗^*p* < 0.05, ^†^*p* < 0.10. All paths control for agentic values, interest in STEM, and societal worth of STEM.

Results, summarized in **Figure 3**, yielded evidence of a significant serial mediation effect, *a_1_^∗^a_2_^∗^b* = 0.01, *SE* = 0.003, bootstrapped *CI*_0.95_ (0.002, 0.02). Gender was a significant predictor of communal values, β = 0.22, *SE* = 0.05, *t*(373) = 4.31, *p* < 0.001; which in turn predicted greater perceived worth of HEED careers, β = 0.11, *SE* = 0.03, *t*(372) = 3.12, *p* = 0.002. Perceiving higher societal worth in HEED, in turn, predicted higher personal interest in adopting HEED careers, β = 0.29, *SE* = 0.07, *t*(371) = 4.19, *p* < 0.001. Results from serial mediation analyses thus suggest that men’s (vs. women’s) relatively lower interest in HEED careers is partially explained through communal values – both through communal values’ relationship to perceived lower societal worth of HEED, but also communal value’s direct relationship to lower interest in HEED careers.

### Discussion

Results from Study 1 suggest that strongly endorsing communal values relates not only to greater interest in adopting HEED careers and less interest in STEM careers (as shown previously by Diekman et al., 2010, 2011), but also predicts perceiving more societal worth in HEED occupations. As expected, men, compared to women, were less interested in pursuing HEED careers themselves and also tended to perceive lower societal value in HEED careers. In turn, gender differences in communal values partially accounted for these gender differences in devaluing HEED on both a personal and societal level. Seeing less societal worth in HEED also partially explained men’s lower interest in HEED. These results suggest that those who care less about nurturing and connection (who are more likely to be men), tend to place less value on roles in society that provide care to others (i.e., HEED). Moreover, given that those who strongly endorsed agentic values tended to assign lower societal worth to HEED (and more to STEM) careers, agency appears to play some role in these evaluations. The lack of gender difference in agency in this large sample, however, made this variable an unlikely potential explanation for gender differences in how these occupations are evaluated.

## Study 2

Although Study 1 provided initial evidence that communion plays a larger role than agency in understanding men’s underrepresentation in (and devaluation of) HEED roles, we were concerned that our abstract measure of agentic values might have obscured relevant facets of the construct. Despite the fact that the gender gap in overall agency is no longer found by all contemporary studies (Diekman et al., 2010), men in most societies remain markedly more competitive than women (Croson and Gneezy, 2009). This gender difference in competitiveness is evident early in development (Gneezy and Rustichini, 2004), and across cultures (Gneezy et al., 2009). In addition, evidence suggests that a competitive mindset lead to less prosocial behavior (Liberman et al., 2004). It is plausible that gender differences in this particular facet of agency offer an alternative explanation for men’s relatively lower interest in and perceived societal worth of HEED careers, because men’s striving for competitiveness could be perceived as incompatible with care-oriented HEED roles (consistent with goal-congruity perspective; Diekman et al., 2017).

Study 2 tested whether possible gender differences in trait competitiveness – as a key component of agency – account for gender differences in HEED role interest and perceived societal worth, over and above the mediational effect of communal values (documented in Study 1). Study 2 was originally designed to test an experimental manipulation of competitiveness, in which participants were randomly assigned to play either a competitively- or a cooperatively framed game that has been used in the past to prime competitive vs. cooperative mindsets (Liberman et al., 2004). Because this manipulation failed to show effects on competitive behavior or self-reported competitiveness, we collapsed across conditions and analyzed the dataset correlationally. Controlling for condition does not change results (see SOM). The strengths and limitations of this approach will be addressed in the Section “General Discussion.”

### Method

#### Participants

We recruited 308 (152 men/156 women) undergraduates from a large Canadian university who participated either for research credit or $10 (*M*_age_ = 20.0, *SD* = 2.23). Participants reported a variety of majors (39.3% from Psychology, 10.7% from other Arts majors, 22.7% from Science majors, 12.7% business, and the rest from other majors) and were predominantly East Asian (52.9%), Caucasian (22.7%), or East Indian (14.0%). Study 2 was run in 2014 with a goal of recruiting a minimum 75 participants per condition and gender. Sensitivity analyses with G^∗^power suggested that this sample was powered to detect a small to medium interaction effect in an ANOVA ($\eta_{p}^{2}$ = 0.025) with 80% power (alpha = 0.05).

#### Procedure

Participants were brought into the lab in pairs, ostensibly for a study examining individual differences in playing games. They completed the study in individual cubicles, thinking that they were playing with a partner in another cubicle. Based on random assignment, they either heard a description of the task as a “cooperation game” played “with a partner” (cooperation condition), or as a “competition game” played “against an opponent.” (competition condition). After learning the rules of the prisoner’s dilemma game (PDG, Liberman et al., 2004), all participants played only a single trial of the game before completing the same measures completed by participants in Study 1 (but on the computer). Because initial analyses revealed that participants were not more likely to choose the competitive option in the PDG as a result of the task description and the manipulation had no effects on other measures, analyses collapsed across this experimental manipulation to instead test our correlational hypotheses parallel to Study 1. More details can be found in the SOM.

#### Measures

As in Study 1, we assessed (in the described order) participants’ interest in HEED (0–100 scale; α = 0.73) and STEM careers (0–100 scale; α = 0.70), participants’ perceptions of societal worth of HEED ($0–$400 per hour scale; α = 0.93) and STEM careers ($0–$400 per hour scale; α = 0.92), and their communal (0–100 scale; α = 0.83) and agentic values (0–100 scale; α = 0.80).^6^

##### Trait competitiveness

Participants self-reported their trait competitiveness after the above described measures on a 9-item measure (α = 0.94; Houston et al., 2002) before completing demographics. Items included positively worded statements (e.g., “I am a competitive individual.”) and negatively worded statements (e.g., “I don’t like competing against other people.”) and were rated on a scale of 0 = “Strongly Disagree” to 100 = “Strongly Agree.”

### Results and Discussion

#### Gender Differences in Outcomes

##### Personal and traits values

A 2 (participant gender) × 3 (value-type: communal, agentic, competitiveness) mixed ANOVA showed the anticipated participant gender by value-type interaction, *F*(1,305) = 32.52, *p* < 0.001, $\eta_{p}^{2}$ = 0.18. Replicating gender differences found by others (Croson and Gneezy, 2009), Bonferroni-corrected pairwise comparisons showed that men scored significantly higher on competitiveness, *p* < 0.001, but significantly lower on communal values, *p* = 0.009, than did women. As in Study 1, men and women did not differ in the extent to which they felt agentic values were important to them, *p* = 0.414. Means, *d*-scores for gender differences, and correlations for all variables can be found in **Table 2**.

**Table 2:** Study 2 descriptive statistics and bivariate correlations.

	1	2	3	4	5	6	7
(1) Communal values	–						
(2) Agentic values	**0.13^∗^**	–					
(3) Competitiveness	0.05	**0.27^∗^**	–				
(4) HEED interest	**0.31^∗^**	**-**0.11	-**0.15^∗^**	–			
(5) HEED value	**0.13^∗^**	0.03	-0.02	**0.16^∗^**	–		
(6) STEM interest	**-**0.06	0.04	0.04	**0.15^∗^**	-0.02	–	
(7) STEM value	0.05	**0.12^∗^**	0.03	0.002	**0.81^∗^**	0.04	–

*M*_men_ (*SD*)	71.76 (12.80)	67.42 (14.43)	66.41 (21.07)	38.27 (20.12)	113.16 (80.47)	41.05 (19.73)	141.81 (84.86)
*M*_women_ (*SD*)	75.48 (12.17)	68.72 (13.36)	51.54 (17.17)	55.55 (16.78)	130.28 (90.46)	34.85 (19.32)	145.46 (90.90)
*d*-score_(men-women)_	**-0.30^∗^**	-0.09	**0.77^∗^**	-**0.93^∗^**	-0.20^†^	**0.32^∗^**	-0.04

*^∗^p < 0.05. Superscripts on d-scores indicate significant level of gender differences from tests reported in text. All scales had a range of 0–100 except the value measures, in which participants expressed value in an hourly pay amount that could vary between $0–$400/hr.*

##### Career interest

As in Study 1, a 2 (participant gender) × 2 (career-type: HEED vs. STEM) mixed ANOVA yielded the predicted interaction, *F*(1,306) = 77.14, *p* < 0.001, $\eta_{p}^{2}$ = 0.20. As expected, simple pairwise comparisons revealed that men reported less interest in HEED careers than did women, *p* < 0.001. Women, in turn, reported less interest in STEM careers than did men, *p* = 0.006. In addition, women favored HEED careers over STEM, *p* < 0.001, whereas men in this sample reported non-significantly lower interest in HEED than in STEM, *p* = 0.145. Perhaps because Study 2 was dominated by students from a HEED-related field (i.e., the psychology participant pool), there was also a general tendency of participants to report more interest in HEED than in STEM careers, *F*(1,306) = 46.47, *p* < 0.001, $\eta_{p}^{2}$ = 0.13.

##### Perceived worth to society

**FIGURE 4:**
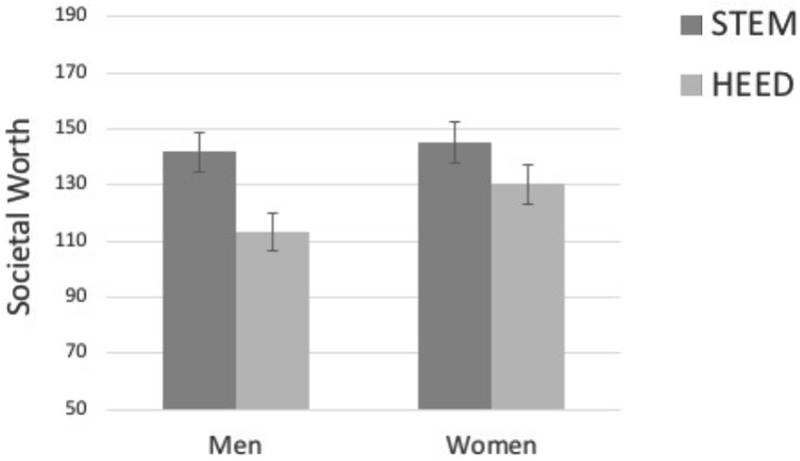
Study 2 perceived societal worth of HEED and STEM by participant gender. Error bars represent standard errors of the mean.

In addition, a 2 (participant gender) × 2 (career-type) mixed ANOVA on perceived societal worth of careers revealed a main effect of career-type, *F*(1,306) = 52.99, *p* < 0.001, $\eta_{p}^{2}$ = 0.15, that was qualified by a significant gender by career interaction, *F*(1,306) = 5.04, *p* = 0.025, $\eta_{p}^{2}$ = 0.02. In this sample, there was no main effect of gender, *F*(1,306) = 1.21, *p* = 0.272, $\eta_{p}^{2}$ = 0.004. As visualized in **Figure 4**, in support of our hypothesis, men assigned marginally less societal worth to HEED careers than did women, *p* = 0.081. Men and women, however, assigned similar levels of societal worth to STEM careers, *p* = 0.716. Parsed differently, both men and women assigned more societal worth to STEM than to HEED, *p*s < 0.001, but the gap was significantly larger for men, *d* = 0.35, than for women, *d* = 0.17.

#### Mediation of Career Attitudes by Personal Values

**FIGURE 5:**
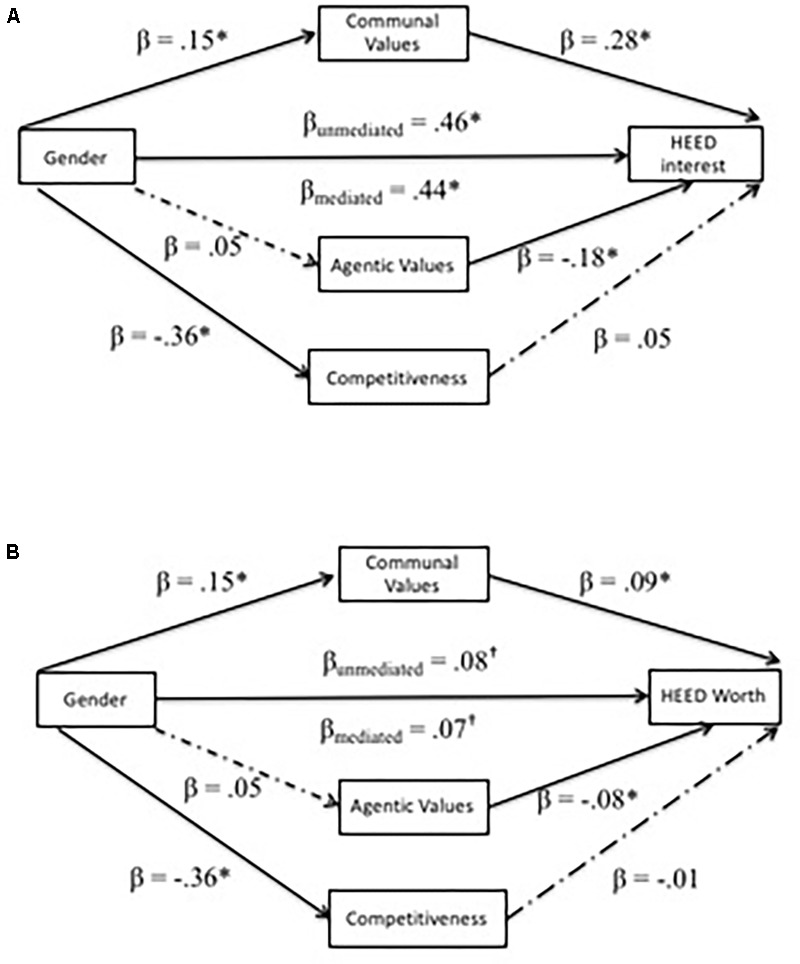
Mediation models for HEED variables in Study 2. **(A,B)** Show mediation models for HEED related outcome variables. All mediators were entered simultaneously for each model. ^∗^*p* < 0.05, ^†^*p* < 0.10.

As in Study 1, we next tested the extent to which in communal values, agentic values, and now also trait competitiveness, predicted evaluations of HEED careers (controlling for gender), that in turn mediate gender differences in HEED perceptions. As before, all possible mediators (communal values, agentic values, trait competitiveness) were entered into the mediational regression model simultaneously to better estimate unique effects, and models also controlled for STEM perceptions.^7^ Results from these analyses are visualized in **Figure 5**.

As documented above, we found gender differences in communal values, β = 0.15, *SE* = 0.06, *t*(306) = 2.61, *p* = 0.010, and trait competitiveness, β = -0.36, *SE* = 0.05, *t*(306) = -6.79, *p* < 0.001, but not agentic values, β = 0.05, *SE* = 0.06, *t*(306) = 0.82, *p* = 0.414. Consistent with the findings from Study 1, endorsement of communal values significantly predicted greater interest in HEED careers (controlling for interest in STEM careers), β = 0.28, *SE* = 0.05, *t*(302) = 5.80, *p* < 0.001, as well as the tendency to assign higher societal worth to HEED careers (controlling for societal worth of STEM careers), β = 0.09, *SE* = 0.03, *t*(302) = 2.60, *p* = 0.010. Over and above communal values and trait competitiveness, agentic values predicted both less interest in HEED, β = -0.18, *SE* = 0.05, *t*(302) = -3.66, *p* < 0.001, and a tendency to perceive lower worth to society in HEED, β = -0.08, *SE* = 0.03, *t*(302) = -2.18, *p* = 0.030. In contrast, despite the previously described gender differences, trait competitiveness did not significantly relate to interest in, β = 0.04, *SE* = 0.05, *t*(302) = 0.66, *p* = 0.512, or societal worth assigned to HEED careers, β = -0.01, *SE* = 0.04, *t*(302) = -0.25, *p* = 0.803.

Finally, bootstrapping analyses to estimate indirect effect sizes yielded significant indirect effects of gender via communal values on both interest in HEED related careers, *a^∗^b* = 0.04, *SE* = 0.02, bootstrapped *CI*_0.95_ (0.01, 0.08), and perceptions of societal worth of HEED careers, *a^∗^b* = 0.01, *SE* = 0.01, bootstrapped *CI*_0.95_ (0.003, 0.03). Given that there was no relationship between competitiveness and these outcomes, analyses yielded no evidence that trait competitiveness mediated either gender differences in HEED interest, *a^∗^b* = -0.01, *SE* = 0.02, bootstrapped *CI*_0.95_ (-0.05, 0.03), or societal worth assigned to HEED careers, *a^∗^b* = 0.003, *SE* = 0.01, bootstrapped *CI*_0.95_ (-0.03, 0.03). Similarly, given the lack of gender differences on agentic values, analyses yielded no evidence that agentic values mediated either gender differences in interest in HEED careers, *a^∗^b* = -0.01, *SE* = 0.01, bootstrapped *CI*_0.95_ (-0.03, 0.01), or societal worth assigned to HEED careers, *a^∗^b* = -0.004, *SE* = 0.01, bootstrapped *CI*_0.95_ (-0.02, 0.004). After entering communal and agentic values, and trait competitiveness (alongside STEM perceptions as control) into these models, gender remained a significant predictor of HEED interest, β = 0.44, *SE* = 0.05, *t*(302) = 8.37, *p* < 0.001, but not of perceived societal worth of HEED, β = 0.07, *SE* = 0.04, *t*(302) = 1.96, *p* = 0.053. These findings further support our hypothesis that relatively lower communal values predict the extent to which individuals in general, and to some extent men in particular, find HEED roles less personally interesting and perceive them as having less worth to society.^8^

##### Are gender differences in HEED interest mediated by communal values and societal worth?

**FIGURE 6:**
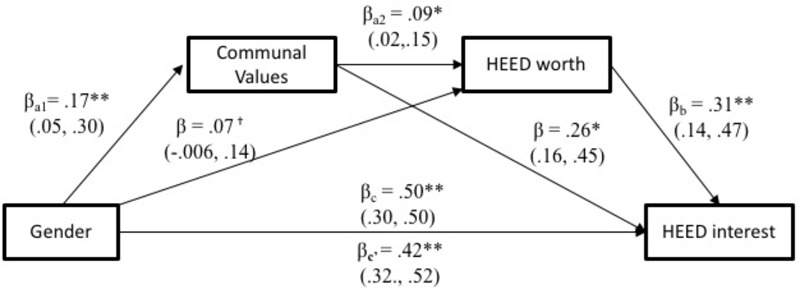
Serial Mediation model for HEED variables in Study 2. All paths control for agentic values, interest in STEM, and societal worth of STEM. ^∗∗^*p* < 0.01, ^∗^*p* < 0.05, ^†^*p* < 0.10. 95% confidence intervals in the brackets.

Lastly, as in Study 1, we conducted serial mediation analyses with the PROCESS macro (Hayes, 2012; model 6) entering gender as the main predictor, communal values as mediator 1, and societal worth of HEED as mediator 2 of a model predicting personal HEED interest as the outcome. Again, all paths controlled for agentic values, trait competitiveness, societal worth of STEM, and interest in STEM. Results, summarized in **Figure 6**, yielded a significant serial mediation effect, a_1_^∗^a_2_^∗^b = 0.004, *SE* = 0.003, bootstrapped *CI*_0.95_ (0.001, 0.01). There were gender differences in communal values, β = 0.18, *SE* = 0.06, *t*(302) = 2.72, *p* = 0.007, which were predictive of higher societal worth perceived in HEED careers, β = 0.09, *SE* = 0.03, *t*(301) = 2.56, *p* = 0.011. Perceiving higher societal worth in HEED, in turn, predicted higher personal interest in taking on HEED careers, β = 0.31, *SE* = 0.08, *t*(300) = 3.72, *p* < 0.001.

### Discussion

Taken together, results of Study 2 replicate findings from Study 1, providing further support for a goal congruity perspective of men’s (and women’s) devaluation of HEED roles. Compared to women, men were less personally interested, and perceived somewhat less societal worth, in HEED careers to the extent they were less likely to have internalized communal values. Consistent with findings from Study 1, further analyses suggest that men’s (vs. women’s) relatively lower interest in HEED careers is partially explained by their lower communal values’ predicting lower societal worth assigned to HEED careers. Results from Study 2 also failed to find any support for the alternative hypothesis that high agency, in general, or high competitiveness, more specifically, can provide better explanations for men and women’s different evaluations of HEED occupations. Irrespective of gender, however, we observed that stronger endorsement of agentic values, over and above gender and trait competitiveness, consistently predicted perceiving HEED careers as contributing *less* worth to society in Studies 1 and 2. Although we replicated a frequently observed gender difference in competitiveness, we found no evidence that more competitive people tend to devalue HEED careers. Together, patterns from the first two studies are in line with our assertion that one factor underlying men’s relatively lower personal interest in and perceived societal worth of careers such as nursing and teaching, is that men are less likely than women to internalize communal values.

## Study 3

Studies 1 and 2, to our knowledge, provide the first evidence for the novel hypothesis that communal values not only predict personal interest in careers but also plays an important role in the broader societal worth people assign to different occupations. There were gender differences in evaluations of HEED careers as having worth to society, but personal communal values consistently predicted these evaluations over and above gender. Given the under-examined nature of this topic, Study 3 was designed to focus more specifically on the extent to which personal values predict both perceptions of the societal worth of HEED careers, and support for efforts to increase HEED salaries (in order to match STEM salaries).

Our first aim was to replicate the relationship between communal values and perceived societal worth of HEED careers using a more rigorous methodology. In Studies 1 and 2, participants expressed the societal worth they perceived in HEED (and STEM) careers as an ideal hourly pay. Whereas this method does provide a meaningful ratio scale, participants’ ratings could easily be skewed by their knowledge of the realistic discrepancies in income or work hours between the different career-types in North America. Because workers in HEED professions (e.g., teaching and nursing) earn lower salaries (Cross and Bagilhole, 2002) and work fewer hours (Statistics Canada, 2017) than comparable male-dominated STEM careers, participants’ ratings of societal worth could be biased by their knowledge of these differences. To address this concern, participants in Study 3 initially rated their perceptions of the *actual* pay and work hours of careers, which allowed us to partial out these ratings from their assessments of *ideal* pay as a measure of perceived worth. In addition, we improved our measures of societal worth by rephrasing the items more clearly, and also by narrowing the focus to careers that clearly require caregiving (i.e., we replaced “human resources manager” and “educational administrator” with “occupational therapist” and “special education teacher”).

The second aim of Study 3 was to examine the relationship between (and gender differences in) communal values and people’s support for policies aimed at increasing HEED salaries to match STEM salaries. Similar to our findings on societal worth in the previous studies, we predicted that those with lower communal values (who also tend to be men) would be less supportive of policies designed to increase HEED salaries. Moreover, we predicted that men would be less likely to support increases in HEED salaries than would women, a gender difference that should be partially accounted for by men’s relatively lower endorsement of communal values, as well as by their tendency to see HEED careers as worth relatively less to society. In testing our hypotheses using a novel operationalization of our key outcome, we also increased the external validity and potential generalizability of our findings.

Our key hypotheses are based on the theoretical assumption that goal congruity processes lead people with stronger communal values to perceive greater societal worth in communal HEED careers, and therefore want HEED careers to be compensated accordingly. Because communal values reflect a more general endorsement of social equality as a prized goal (Schwartz and Bilsky, 1987), one would expect that individuals who are more communal should also be more supportive of increasing gender balance (i.e., a form of equality) in any field (HEED or STEM). We thus tested whether communal values (and gender differences in them) would predict participants’ support for social action aiming to increase the gender balance in general (i.e., average support for increasing gender balance for both HEED and STEM). However, we also explored whether communal values uniquely predicted support for increasing gender balance in HEED *over and above* support for gender balance in STEM.

Lastly, in Studies 1 and 2, we found little evidence that men’s evaluations of HEED careers are explained by the value they placed on agency or a desire to be competitive. A final aim of Study 3 was to test a new alternative hypothesis that men’s relatively lower worth placed on HEED careers is instead (or additionally) predicted by their valuation of material wealth. If men value money more than do women, then this prioritization of money could reasonably predict their more positive judgment of STEM careers, which drive economic growth (Cooke, 2002), over HEED careers which are traditionally publicly funded and pay lower salaries (Cross and Bagilhole, 2002; Bagilhole and Cross, 2006).

### Method

#### Participants

A total of 307 undergraduate students completed the study in individual cubicles in the lab (run in 2016). This number was higher than our *a priori* target of 280 because we oversampled to account for exclusions due to failed attention checks. Our target sample was calculated by estimating the sample size needed to obtain 85% power to find an indirect effect equal to the average effect size we found in Studies 1 and 2. We excluded 15 participants who failed basic attention checks indicating that they randomly chose answers (e.g., “If you are paying attention, please select option two.”) and one participant who did not identify as either male or female, leaving a final sample of 291 (146 men/145 women). Participants were on average 20.06 years old (*SD* = 2.34) and were 1st (27.5%), 2nd (32%) or 3rd (25.1%) year students in Psychology (38.1%), other Arts majors (20.3%) and other Science majors (15.1%). Participants were predominantly East Asian (47.80%) or White (26.80%).

#### Materials and Procedure

##### Personal values

As in the previous studies, participants began by rating the extent to which seven *communal values* (α = 0.85) and seven *agentic values* (α = 0.79) were personally important to them. Embedded with these values, participants in Study 3 also rated two items (“Money”; “Wealth,” *r* = 0.85, *p* < 0.001) which were combined to assess participants’ endorsement of *material values*. All ratings were made on a scale of 1 (Not at all important) to 9 (Extremely Important)^9^ and all values were presented in randomized order.

##### Perceived career attributes

Before rating the perceived societal worth of each career, participants were asked to estimate the *real salary*, rated on a scale of ‘$0 per hour’ to ‘$150 per hour’, and then the *weekly work hours*, rated on a scale of “0 h a week” to ‘90 h a week,’ for each HEED career (α_salary_ = 0.88, α_hours_ = 0.79), STEM career (α_salary_ = 0.93, α_hours_ = 0.87).

##### Worth to society

We updated the phrasing of this item to increase clarity. Participants in Study 3 were asked to “assign a dollar amount to represent what you think each of the following careers should be paid based on their worth TO THE FUNCTIONING OF SOCIETY” (added text in all caps). In this way, participants rated the worth to society of five HEED careers (nurse, social worker, special education teacher, occupational therapist, and elementary school teacher; α = 0.94) and five STEM careers (computer systems architect, industrial engineer, mechanical engineer, architect and software developer; α = 0.94).^10^ All careers were presented in a randomized order. Ratings were made on a continuous slider scale with the anchors $0 to $150 per hour. This range was updated to more closely match the actual average pay of all the occupations used according to data from the Canadian government (Government Canada, 2015) with 20% added to the highest average hourly pay.

##### Support for change

After making their ratings of specific careers, participants completed three measures of support for social change in regards to HEED and STEM that served as three novel outcome variables: (1) support for HEED salary increases (to match salaries in STEM), (2) support for increasing the gender balance in HEED, and (3) support increasing the gender balance in STEM. Given that we were most interested in HEED perceptions, scales were always presented in this order. All ratings were made on a scale of 1 (strongly disagree) to 7 (strongly agree) and items were randomized *within* each outcome variable.

##### Support for salary increases

Participants first read a paragraph describing that employees in HEED careers are typically paid less than those in STEM careers, despite requiring similar amounts of education and work hours. Participants then rated their agreement with seven statements on the value of policies and governmental action aimed at increasing HEED salaries to match salaries in STEM (e.g., “It would be fair to increase salaries for occupations such as nursing, teaching, and social work until they become similar to salaries in engineering and technology related occupations.” and “We do NOT need to try to increase the pay of nurses, teachers and social workers to match those of engineers and computer scientists.”; α = 0.91). The full measure is provided in **Appendix 1**.

##### Support for increasing gender balance

Next, participants completed two measures that assessed the extent to which they support making efforts toward equal gender representation in HEED careers and STEM careers. First, participants read about gender imbalances in HEED and STEM occupations before rating the extent to which they agree with 10 statements about *support for gender balance* in HEED (α = 0.93; e.g., “Professions such as nursing, teaching, and social work would be enhanced with a more equal distribution of men and women” and “Policies should be enacted to encourage hiring more men in jobs where they are fewer in number, such as nursing, teaching, and social work”). Next participants rated 10 parallel statements about support of gender balance in STEM (α = 0.94). To create and index of *general support for gender balance* we first z-scored all items for support of gender balance HEED and in STEM and then averaged these 20 items into the overall index (α = 0.95).

##### Demographics and exploratory variables

Along with several exploratory variables assessing participants’ perceptions of the ideal priorities of a society and compatibility of communal and agentic values, participants completed a standard demographic questionnaire including age, gender, year in school, major, ethnicity, SES, ethnicity, marital status and political orientation. In addition, participants answered two open-ended questions designed to assess what they thought the study was about and whether they had any idea about our specific hypothesis. All measures are listed in the SOM.

### Results and Discussion

#### Gender Differences

##### Personal values

Descriptive statistics, correlations, and gender differences for all variables in Study 3 are summarized in **Table 3**. As in the previous studies, a 2 (participant gender) × 3 (value-type: communal, agentic, material) mixed ANOVA yielded the expected interaction, *F*(1,288) = 5.08, *p* = 0.007, $\eta_{p}^{2}$ = 0.03. Bonferroni-corrected pairwise comparisons suggested that, once again, men reported lower communal values than did women, *p* = 0.033, but men and women showed comparable levels of both agentic values, *p* = 0.152, and material values, *p* = 0.911. Comparisons within gender (Bonferroni-corrected) suggested that men endorsed material and agentic values at similar levels, *p* = 0.556, whereas women endorsed material values significantly more than broad agentic values, *p* = 0.006. Both men and women, however, reported valuing communion more than either of the two other values, *p*s < 0.003.

**Table 3:** Study 3 key variable descriptives and bivariate correlations.

	1	2	3	4	5	6	7	8	9	10	11	12	13
(1) Communal values	-												
(2) Agentic values	0.12#	-											
(3) Money values	-**0.12^∗^**	**0.68^∗^**	-										
(4) HEED worth	0.09	-0.04	-0.09	-									
(5) HEED salary	-0.03	0.01	-0.01	**0.63^∗^**	-								
(6) HEED hours	-0.03	-0.08	-0.08	**0.38^∗^**	**0.30^∗^**	-							
(7) STEM worth	-0.03	0.06	0.09	**0.73^∗^**	**0.63^∗^**	**0.28^∗^**	-						
(8) STEM salary	0.03	-0.03	0.01	**0.61^∗^**	**0.67^∗^**	**0.19^∗^**	**0.76^∗^**	-					
(9) STEM hours	-0.09	-0.02	-0.05	**0.35^∗^**	**0.31^∗^**	**0.58^∗^**	**0.47^∗^**	**0.30^∗^**	-				
(10) Support pay increase	**0.25^∗^**	-**0.21^∗^**	-**0.29^∗^**	**0.16^∗^**	0.04	0.03	-**0.14^∗^**	-0.04	-**0.18^∗^**	-			
(11) HEED gender balance	**0.23^∗^**	-0.01	-0.09	**0.14^∗^**	0.08	0.02	0.06	**0.12^∗^**	-0.05	**0.35^∗^**	-		
(12) STEM gender balance	**0.22^∗^**	-0.06	-0.10#	**0.14^∗^**	0.05	-0.01	0.02	**0.12^∗^**	-0.09	**0.43^∗^**	**0.69^∗^**	-	
(13) General gender balance	**0.24^∗^**	-0.04	-0.10	**0.15^∗^**	0.07	0.004	0.04	**0.13^∗^**	-0.07	**0.43^∗^**	**0.92^∗^**	**0.92^∗^**	-

*M*_men_ (*SD*)	7.03 (1.11)	6.32 (1.24)	6.45 (1.73)	53.88 (23.09)	35.67 (14.00)	43.93 (7.78)	57.15 (22.95)	56.95 (24.23)	46.36 (9.41)	4.57 (1.14)	4.28 (1.11)	4.55 (1.17)	-0.33 (0.71)
*M*_Women_ (*SD*)	7.31 (1.11)	6.12 (1.08)	6.43 (1.46)	58.26 (27.77)	41.94 (17.53)	45.11 (8.69)	57.48 (24.43)	65.88 (26.60)	45.01 (9.03)	5.37 (1.11)	5.08 (0.93)	5.60 (0.93)	0.33 (0.59)
*d*-score*_(men__-women)_*	**-0.25^∗^**	0.17	0.01	-0.17^†^	-**0.40^∗^**	-0.14	-0.01	-**0.35^∗^**	0.15	-**0.71^∗^**	-**0.78^∗^**	-**0.99^∗^**	-**1.01^∗^**

*^∗^p < 0.05. Superscripts on d-scores indicate significant level of gender differences from tests reported in text. All scales had a range of 1–7 except the measures of worth, actual pay, and work hours. Means and SD for columns 4, 7, 10, 11, 12 are marginal estimates accounting for reported covariates, gender differences are thus smaller than raw differences.*

##### Perceived career attributes

A goal in this study was to better disentangle perceptions of societal worth of HEED from participants’ estimates of the real salary and work hours of HEED and STEM careers in the current labor market. A 2 (participant gender) × 2 (career-type) mixed ANOVA on estimated *real salary* revealed only main effects of career-type, *F*(1,289) = 400.88, *p* = <0.001, $\eta_{p}^{2}$ = 0.58, and of gender, *F*(1,289) = 11.77, *p* = 0.001, $\eta_{p}^{2}$ = 0.04, but no gender by career-type interaction, *F*(1,289) = 1.37, *p* = 0.244, $\eta_{p}^{2}$ = 0.01. These effects suggested that participants correctly perceived that STEM careers pay higher wages than HEED careers, but also that women generally reported higher salary estimates for both career-types than did men.

A 2 (participant gender) × 2 (career-type) mixed ANOVA on *perceived work hours* revealed a main effect of career-type, *F*(1,289) = 6.28, *p* = 0.013, $\eta_{p}^{2}$ = 0.02, but no effect of gender, *F*(1,289) = 0.01, *p* = 0.928, $\eta_{p}^{2}$ < 0.001. Importantly these effects were qualified by a significant interaction, *F*(1,289) = 7.38, *p* = 0.007, $\eta_{p}^{2}$ = 0.03. Simple pairwise comparisons showed that there were no significant gender differences in perceived work hours for either HEED, *p* = 0.220, or STEM careers, *p* = 0.212. However, whereas women estimated similar work hours for STEM and HEED, *p* = 0.878, men thought that employees in STEM careers worked significantly longer hours than those in HEED careers, *p* < 0.001.

##### Perceived worth to society

**FIGURE 7:**
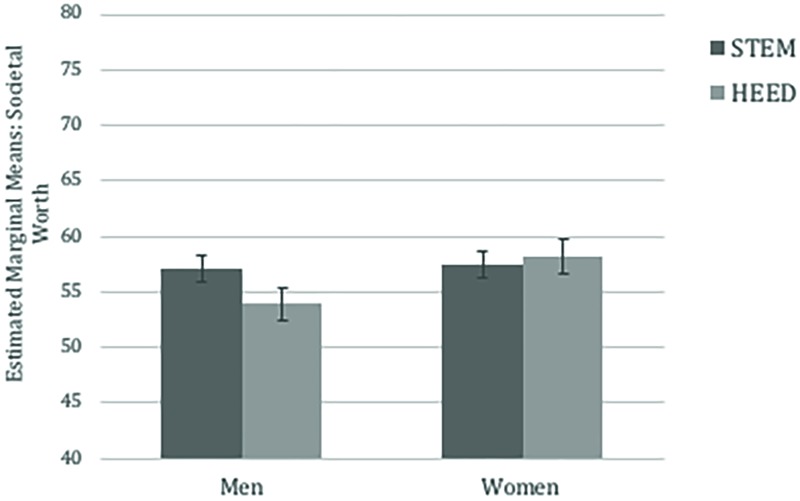
Study 3 perceived societal worth of HEED and STEM by participants gender. Error bars represent standard errors of the mean. Graphed means are marginal estimates accounting for perceived pay and work hours in both occupation types.

In line with hypotheses, people’s estimates of HEED careers’ actual salary, *r* = 0.63, *p* < 0.001, and work hours, *r* = 0.38, <0.001, were both positively related to greater perceived societal worth in HEED careers. To test whether gender differences in the worth of HEED careers were robust to these estimates of the actual labor market, we analyzed participants’ societal worth ratings in a 2 (participant gender) × 2 (career-type) mixed analysis of covariance (ANCOVA) controlling for estimates of real salary and work hours for both STEM and HEED careers. Adjusted mean estimates from these analyses are displayed in **Figure 7**. Consistent with hypotheses, there was a marginal gender × career-type interaction, *F*(1,285) = 3.77, *p* = 0.053, $\eta_{p}^{2}$ = 0.01. The main effects of career-type, *F*(1,285) = 0.88, *p* = 0.348, $\eta_{p}^{2}$ = 0.003, and gender were not significant, *F*(1,285) = 1.92, *p* = 0.167, $\eta_{p}^{2}$ = 0.01. As in Study 2, simple pairwise comparisons showed that although women perceived STEM and HEED careers to have similar societal worth, *p* = 0.591, men perceived STEM to have greater societal worth than HEED, *p* = 0.024. In addition, men tended to undervalue HEED careers compared to women, *p* = 0.053, whereas men and women assigned similar societal worth to STEM roles, *p* = 0.846. The fact that the size of these gender differences was reduced by controlling for participants’ estimates of current salary and work hours suggests that the perceived worth ratings were, as we had suspected in the previous studies, somewhat contaminated with perceptions of the real labor market. However, even after accounting for the extent to which perceived worth is also tied to also perceiving HEED careers to actually work less hours and earn lower salaries, men perceive significantly less worth in HEED compared to STEM careers.^11^

##### Support for social change

In addition to their perceptions of the societal worth of HEED and STEM, participants also rated their support for pay increases in HEED (to match those of STEM) and support for gender balance in both HEED and STEM careers. For support for HEED salary increases, we conducted a one-way ANCOVA comparing participants’ support for that type of social change, controlling for estimated real salary and work hours in both HEED and STEM careers. Results for HEED salary increase suggested that, as we expected, men tended to support increases in HEED salaries significantly less than did women, *F*(1,285) = 35.17, *p* < 0.001, $\eta_{p}^{2}$ = 0.11^12^. Estimated work hours in HEED, *F*(1,285) = 3.79, *p* = 0.055, $\eta_{p}^{2}$ = 0.01; and STEM, *F*(1,285) = 9.87, *p* = 0.002, $\eta_{p}^{2}$ = 0.03; were marginal and significant covariates in the model, respectively; whereas pay perceptions in HEED and STEM, were not, *F* < 2.40, *p*s > 0.120. These results suggest that gender differences in support for HEED salary increases are robust to controlling for labor market perceptions.

Furthermore, a 2 (gender) × 2 (career-type) mixed ANCOVA on support for attaining gender balance within each career-type (again controlling for career perceptions) yielded a main effect of gender, *F*(1,285) = 65, *p* < 0.001, $\eta_{p}^{2}$ = 0.19; that was qualified by a significant participant gender × career-type interaction, *F*(1,285) = 2.08, *p* = 0.024, $\eta_{p}^{2}$ = 0.02. Women were more supportive than men of promoting gender balance in HEED as well as in STEM careers, all *p*s < 0.001. Furthermore, both men and women supported increasing gender balance more in STEM than in HEED, all *p*s < 0.001, although this difference was significantly larger for women, *d* = 0.56, than for men, *d* = 0.24.

#### Do Gender Differences in Values Predict Support for Social Change?

**FIGURE 8:**
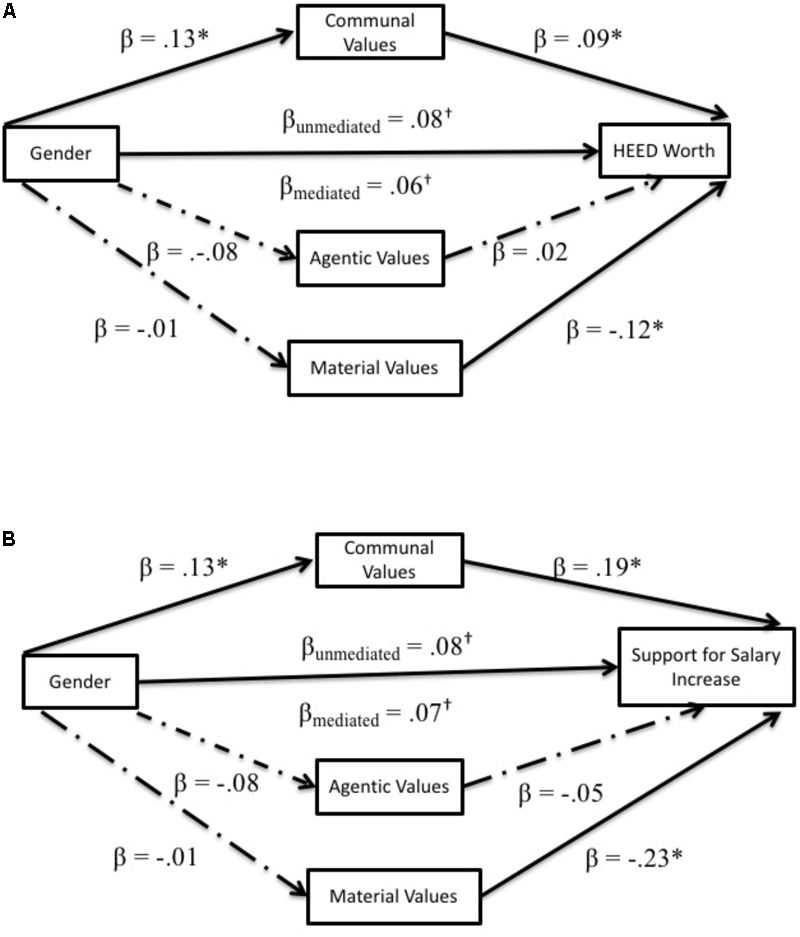
Mediation models for HEED variables in Study 2. **(A,B)** Show mediation models for HEED related outcome variables. All mediators were entered simultaneously for each model. ^∗^*p* < 0.05, ^†^*p* < 0.10.

Our primary goal in Study 3 was to test our hypotheses that communal values would predict both *societal worth of HEED* and *support for HEED salary increases*. We tested these relationships controlling for participants’ perceptions of salary and work hours in the real labor market. We also examined material values as an alternative predictor of these outcomes. In mediational analyses using the PROCESS macro (Hayes, 2012; Model 4), we first regressed societal worth of HEED and support for HEED salary increases (in separately models) onto *communal values, agentic values, and material values* as simultaneous mediators of the observed gender difference on each variable. As before, analyses with perceived societal worth of HEED as an outcome also controlled for the perceived societal worth of STEM careers, but all analyses also controlled for perceived real salary and work hours of the outcome career-type.^13^ Results are summarized in **Figure 8**.

Replicating the results of the prior two studies, communal values predicted a tendency to assign significantly greater societal worth to HEED careers, β = 0.09, *SE* = 0.04, *t*(281) = 2.27, *p* = 0.034, as well as stronger support for HEED salary increases, β = 0.19, *SE* = 0.05, *t*(282) = 3.45, *p* < 0.001. With material values now in the model, agentic values did not uniquely predict perceived societal worth of HEED or support of salary increases, βs < 0.06, *t* < 0.74, *p* > 0.457. However, the endorsement of material values did significantly predict both lower ratings of societal worth in HEED careers, β = -0.12, *SE* = 0.05, *t*(282) = -2.40, *p* = 0.017, and less support for increasing HEED salaries, β = -0.23, *SE* = 0.07, *t*(281) = -3.19, *p* = 0.002.

Given men’s tendency to report lower communal values than did women, bootstrapping analyses revealed significant indirect effects of gender thorough communal values on societal worth of HEED, *a^∗^b* = 0.01, *SE* = 0.01, bootstrapped *CI*_0.95_ (0.001, 0.03), as well as support for HEED salary increases, *a^∗^b* = 0.02, *SE* = 0.01, bootstrapped *CI*_0.95_ (0.004, 0.06). Given the absence of any gender differences in agentic and material values, indirect effects through these variables were non-significant, all *a^∗^b*s < 0.01, *p*s > 0.05. These effects provide evidence that men’s relatively lower communal value endorsement can partly account not only for their different evaluations of HEED roles, but might also explain why men, compared to women, are less concerned about efforts to promote higher salaries paid to HEED careers. Moreover, these results address concerns that the previously observed effects might be biased by participants’ awareness of the actual salary and work hours of these careers on the labor market.

In additional secondary analyses, we tested communal, agentic, and material values as simultaneous mediators of (a) the gender difference in *general* support for gender balance (averaged responses to both STEM and HEED questions), as well as (b) the gender in difference in supporting increased gender balance specifically in *HEED* (controlling for support of gender balance in STEM). As before, all analyses also control for the estimated real salary and work hours of both HEED and STEM in the outcome. Analyses on general support for gender balance revealed that communal values did significantly relate to greater support for increasing overall gender balance in careers, β = 0.16, *SE* = 0.04, *t*(282) = 2.85, *p* = 0.005, and previously described gender difference in communal values thus accounted for a significant proportion of the gender difference in support of gender balance, *a^∗^b* = 0.02, *SE* = 0.01, bootstrapped *CI*_0.95_ (0.002, 0.06). After accounting for gender differences in communal values, female-gender still predicted higher support of general gender balance, β = 0.43, *SE* = 0.04, *t*(282) = 7.82, *p* < 0.001. In contrast, analyses revealed that none of the three personal values significantly predicted support for gender balance in HEED specifically, after controlling for gender balance in STEM, βs < 0.08, *t*s < 1.35, *p*s > 0.180, and thus none of the indirect effects were significant, *a^∗^b*s < 0.006.^14^ Thus, those who are more communal support reducing occupational segregation in both male and female-dominated roles, not only in HEED.

#### Does Societal Worth Mediate Gender Difference in Support for Salary Increases in HEED?

**FIGURE 9:**
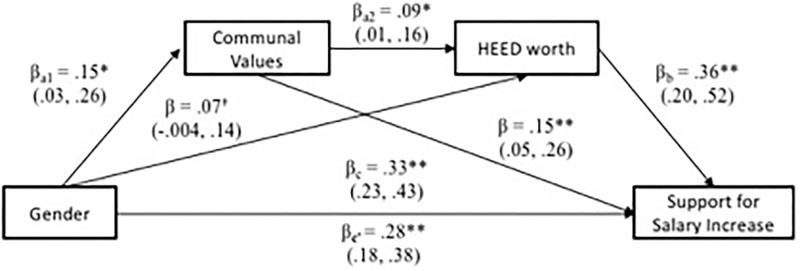
Serial mediation model predicting support for salary increase in Study 3. ^∗∗^*p* < 0.01, ^∗^*p* < 0.05, ^†^*p* < 0.10.

Given that communal values predicted both the perceived societal worth of HEED as well as support of salary increases in HEED, a final analysis examined whether the gender difference in support for salary increases was mediated by the perceived societal worth of HEED. To test this, we conducted serial mediation analyses with the PROCESS macro (Hayes, 2012; Model 6), entering gender as the independent variable, communal values as the first mediator, and societal worth of HEED as the second mediator in predicting support for salary increases in HEED careers as the outcome. Again, all paths controlled for agentic values, material values, and perceived pay and work hours for HEED and STEM, as well as societal worth of STEM. Results of bootstrapping analyses, summarized in **Figure 9**, revealed a significant serial mediation (gender → communal values → societal worth of HEED → Support for salary increases), *a_1_^∗^a_2_^∗^b* = 0.005, *SE* = 0.003, *CI*_0.95_ (0.001, 0.01). In addition, results suggested that both simple indirect effects also remained significant; (1) gender → communal values → Support for salary increases, *a_1_^∗^b* = 0.02, *SE* = 0.01, *CI*_0.95_ (0.01, 0.05), and (2) gender → societal worth of HEED → Support for salary increases, *a_2_^∗^b* = 0.03, *SE* = 0.01, *CI*_0.95_ (0.002, 0.06). These results suggest that gender differences in communal values and perceived societal worth of HEED, combined, explain 15% of the variance in men’s tendency to be less supportive of increases in HEED salaries than are women.

## General Discussion

Despite their importance to the well-being of societies, HEED careers are devalued both on a personal and a societal level, perhaps especially by men. The first aim of the current research was to apply the goal congruity perspective – the idea that we evaluate careers based on how they fit our personal values (Diekman et al., 2017) – to understand men’s relative lack of personal interest in adopting careers in healthcare and education. Studies 1 and 2 provided support for our prediction that men’s relatively lower communal values partially accounted for men’s (compared to women’s) lack of interest in HEED careers. Just as past research suggests that women are deterred from STEM careers due to perceiving them as incompatible with their strong communal values (see Diekman et al., 2017), these findings lend support to the assertion that men’s relatively lower internalization of communal values leads them to see communal careers in healthcare and teaching as less attractive career options.

A more novel contribution of the current research was to extend the tenants of the goal congruity perspective to understand men’s, but also women’s, tendency to devalue HEED careers. In all three studies, we found that HEED (compared to STEM) careers are seen as providing less worth to society, in line with predictions derived from status-value theory. As expected, men devalue HEED careers more than do women – they perceive HEED as having somewhat less societal worth (all studies) and are significantly less supportive of increasing HEED salaries (Study 3). In addition, evidence suggests that these gender differences can be explained by goal congruity processes. Men’s, compared to women’s, relatively lower communal values partially accounted for their tendency to perceive lower societal worth and to be less supportive of salary increases for HEED. In turn, these perceptions of societal worth (as predicted by their lower communal values), also predict men’s relatively low interest in taking on HEED careers in the future.

In addition to explaining gender differences in HEED perceptions, our results have implications for the broader way that goal congruity processes shape people’s perceptions of what roles have worth. Whereas actual HEED and STEM salaries are realistically shaped by structural factors – such as their disproportionate representation in the public vs. private sector, respectively – our evidence suggests that men’s and women’s desire to afford certain careers with higher salaries is predicted, at least in part, by the basic values they internalize. Even when controlling for perceptions of current labor market characteristics such as actual salary and work hours (Study 3), individual differences in communal values consistently predict perceptions of the societal worth and support for salary increases in HEED careers – over and above perceiver gender. These novel findings suggest that the abstract values we espouse can directly account for our willingness to take on certain careers ourselves and even predict which careers we perceive as worthwhile to society in general.

Although we focused our investigation on the role of communal values in the gendered perception of HEED careers, we also assessed whether other dimensions of individual differences – broad agentic values, or trait competitiveness and material values – might relate to men’s and women’s tendency to devalue HEED careers at a personal and/or societal level. Our findings suggest that none of these additional value dimensions can account for *gender differences* in perceptions of HEED careers. Yet, we find some evidence that, over and above gender, individuals who value agency more highly, and specifically those who value material gains, tend to perceive HEED careers as having lower societal worth. This is especially meaningful since historical data trends show a general increase in agentic self-evaluations (i.e., achievement motivations) among both men and women in America in the last 40 years (Twenge et al., 2012). Whereas future research should aim to replicate these effects, our work provides preliminary evidence that valuing independence, status, and especially wealth is linked to the perception that communally oriented HEED careers provide less worth to society than STEM careers.

### Limitations and Future Directions

Whereas the current research is, to our knowledge, the first to apply a goal congruity lens to men’s broad evaluations of HEED careers, our research methodology has important limitations. First, the correlational nature of our analyses prevents strong conclusions that communal values *cause* evaluations of HEED careers. However, given the conceptualization of values as relatively stable (Trapnell and Paulhus, 2012), it’s somewhat less likely that evaluations of specific HEED roles cause the broader communal values one endorses. In addition, one possible limitation of the current research is that Study 2 was initially designed as an experimental test of the effects of competitiveness on career evaluations. We adapted a manipulation that, in past studies (Liberman et al., 2004), had successfully primed competitive vs. cooperative mindsets in a prisoner’s dilemma game. As detailed in the SOM, this manipulation failed to show any effects on participants’ choice of how to play the game. It is unclear why we failed to find effects of this manipulation on provoking a competitive mindset or behavior. Yet, Study 2 is well-powered, like all other studies in the paper, and closely replicates results from Study 1 with almost identical measures. In addition, results remain the unchanged when controlling for condition (see SOM), further assuaging any potential concerns that this failed manipulation eroded our ability to test correlational hypotheses.

Our conclusions are further limited by the nature of our measures. Whereas we took care to design measures that were face-valid and intuitive to our participants, our measures ask participants to make relatively explicit judgments about careers which may or may not predict their actual behavior or decision making. First, we asked participants to assign an ideal salary based on a career’s value to society, but the construct *value or worth* can be construed in a number of ways (e.g., value to the survival vs. the productivity of society). Future research should consider different operationalizations of perceived societal worth. Second, future research might also use behavioral measures of career evaluations (e.g., actual donations to career-training programs) to assess the realistic consequences of people’s evaluations. Third, given that people tend to have poor introspective insight for their motivations (Nisbett and Wilson, 1977), and that reporting high levels of communal values is socially desirable (Fiske et al., 2007; Fiske, 2018), future researchers might consider measuring communal value endorsement with more indirect or implicit measures.

Moreover, despite our attempts to rule out possible alternative explanations for our findings, such as current labor market conditions biasing perceptions of HEED careers, the correlational nature of our analyses prevents us from conclusively ruling out other forces that might play into the devaluation of HEED roles. For example, both social role theory (Eagly, 1987; Eagly and Wood, 2012) and the status-value asymmetry perspective (Schmader et al., 2001) would suggest that the mere fact that women are overrepresented in HEED can itself influence how these careers are perceived. Future research should aim to disentangle the effects of gender representation in a given career from the effects that a career’s value-affordances have on its perceived societal worth, perhaps using novel or ambiguous occupational descriptions.

On a related note, given that we only provide correlational evidence, future research should also consider experimental tests of the relationship between personal values and HEED evaluations. Even if the relationship between individual differences in communal values and perceptions of HEED is not spuriously caused by a third variable, it is unclear whether or not increasing men’s communal values could directly increase perceived worth of HEED careers. Men, in most societies, face rigid masculine gender roles norms and, consequently, are wary of transgressing such norms (Vandello and Bosson, 2013). Thus, theorists have suggested that gender role norms (Croft et al., 2015) and especially the expectation to become the primary breadwinner (Diekman et al., 2017) might constrain men’s career aspirations and evaluations, even if a given career would match their personal values. Future research might explore different avenues for creating a better match between HEED roles and men’s internal values – e.g., by increasing men’s communal values directly, or reframing the value-affordances of HEED – in conjunction with efforts to remove external normative pressures for men to devalue HEED careers.

Given our restricted sample of North American undergraduates, the generalizability of our results also remains an open question. Our findings could potentially provide a framework for understanding cultural differences in the status and pay of careers, because not all cultures undervalue their healthcare workers and teachers. In Finland, for example, teaching ranks among the most highly respected and desirable occupations (Ahonen and Rantala, 2001). Past research suggests that in collectivistic cultures, both men and women see themselves as more communal (Cuddy et al., 2015). In light of our findings, future research should sample more diverse populations, and examine whether cultural differences in men’s communal values might explain the status and pay of HEED careers differently by country or cultural backdrop.

### Implications

Our findings lead to new directions for understanding how we evaluate male- vs. female-stereotypic careers. In the interview quoted at the beginning of this article, Anne-Marie Slaughter suggests that true gender equality will only become feasible if we can encourage both men and women to perceive communal roles as more worthwhile. Our findings highlight that men’s and women’s basic communal value endorsement is related to such perceptions of HEED as worthwhile. Because previous research suggests that especially men can confer status onto careers (Reskin, 1988; Schmader et al., 2001; Major et al., 2002) and are seen as the standard for societal ideals (Cuddy et al., 2015), elevating communal activities in the eyes of men might be the first step toward increasing the status of vital HEED careers.

## Ethics Statement

All studies were conducted after review and approval from the Behavioural Research Ethics Board of the University of British Columbia and in line with current guidelines of the Canadian Tri-Council Policy Statement. Studies were run under approved applications H10-03173 and H15-00087. All subjects completed an informed consent and were informed of any deception after the study.

## Author Contributions

KB and TS worked together conceptualized hypotheses and study design. KB spearheaded data collection and analyzed data under the supervision of TS. AC helped conceptualize Study 3 and provided critical feedback and edits throughout the data analysis and writing process.

## Conflict of Interest Statement

The authors declare that the research was conducted in the absence of any commercial or financial relationships that could be construed as a potential conflict of interest.

## Footnotes

1 Study 1 collapses across data from five versions of the same survey measures collected at the same time by the same research assistants. These versions simply varied item-order in an attempt to subtly prime a communal mindset, but initial analyses revealed no differences by order.

2 An additional item, “lawyer” was excluded from the STEM scale given a low item-total correlation, *r* = 0.11, compared to at least 0.20 for all other items, and the lack of gender-imbalance in law careers (Bureau of Labor Statistics, 2017). An item “homemaker” was excluded from the HEED scale given our focus on paid careers.

3 An exploratory maximum likelihood factor analysis with direct oblimin rotation confirmed that participants’ ratings of societal worth formed two factors that, although positively correlated, *r* = 0.55, included ratings of HEED careers on one factor (56.11% of variance) and ratings of STEM careers on the second factor (13.40% of variance).

4 Communal and agentic values were positively correlated, *r*(378) = 0.15, *p* = 0.003. Both for HEED interest and societal worth of HEED, communal values remain a significant mediator of this relationship when agentic values and STEM ratings are NOT included. See **Supplementary Online Materials** (SOM) for these analyses.

5 Though not our focus, we conducted parallel analyses on evaluations of STEM roles. These mediational models (summarized in SOM) revealed that communal values predicted lower interest in STEM careers, suggest that communal values significantly mediated gender differences in STEM interest and predicted marginally lower societal worth perceived in STEM careers (given an absence of a gender difference in perceived societal worth of STEM).

6 We removed the item “competition” from the agentic values composite because it was highly correlated with trait competitiveness, *r* = 0.70, *p* < 0.001, and we aimed to disentangle these constructs. Results are unchanged when including this item.

7 Both communal values, *r*(308) = 0.15, *p* = 0.009, and competitiveness, *r*(308) = 0.41, *p* < 0.001, correlated positively with agency. As documented in the SOM, all indirect effects through communal values remain significant when adding experimental condition as control variable, and when removing agentic values, trait competitiveness, and control variables from all models.

8 Consistent with Study 1 and past research (Diekman et al., 2017), analyses summarized in the SOM revealed that communal values predicted less interest in STEM related careers, thereby mediating gender differences in STEM interest. Gender differences were absent for perceived societal worth of STEM, and communal values were only marginally related to lower societal worth of STEM.

9 We changed the response format given that we switched our survey program to Qualtrics for the last study, and found this response format more visually intuitive.

10 Interspersed between these careers, were two domestic roles (homemaker and stay-at-home parent; *r* = 0.70, *p* < 0.001) and five careers which tend to be gender balanced (Bureau of Labor Statistics, 2017; retail manager, accountant, business account manager, financial analyst and marketing manager; α = 0.94). These items were not central to our hypotheses and will not be discussed further in this paper.

11 Without the covariates entered, gender differences show very similar patterns to Studies 1 and 2. The notable difference is that, without covariates, the gender × career-type interaction is significant, *p* = 0.010, and the gender difference in societal worth of HEED is also significant, *p* = 0.044.

12 This gender difference is similar, $\eta_{p}^{2}$ = 0.13, without covariates in the model.

13 The indirect effect suggesting that communal values is a mediator of gender differences societal worth of HEED is robust to removing all covariates except perceived societal worth of STEM. When societal worth of STEM is removed, the relationship between communal values and HEED worth is of similar magnitude but non-significant, β = 0.07, *SE* = 0.06, *t*(289) = 1.16, *p* = 0.247.

14 Parallel analyses with societal worth of STEM as outcome suggested that communal values did not predict societal worth of STEM careers after accounting for covariates. Despite the absence of gender difference in *material values*, having stronger material values did, however, predict greater perceived societal worth of STEM. Parallel analyses on gender equality support for STEM also showed no significant relationships or indirect effects through any of the value variables. Details in the SOM.

## Appendix 1

### Support for Salary Increases

Not all careers in society are paid the same, even if they require very similar levels of education and work hours. Careers in healthcare, teaching and social work currently pay less than careers in technology and engineering that require similar levels of education. Please indicate the degree to which YOU agree or disagree with the following statements?

(1 D I strongly disagree, to 7 D I strongly agree)

1. The government should enact policies to encourage an increase in pay in occupations such as nursing, teaching, and social work so that their pay will match levels of pay in technology and engineering.
2. Governments should commit funding toward increasing salaries in occupations such as nursing, teaching, and social work to match salaries in technology and engineering.
3. It would be fair to increase salaries for occupations such as nursing, teaching, and social work until they become similar to salaries in engineering and technology related occupations.
4. Greater pay equality for occupations such as nursing, teaching and social work would be beneficial to society as a whole.
5. The different levels of pay we currently see when comparing the fields of nursing, teaching and social work to the fields of engineering and technology are justified. (R)
6. It is sensible that those with occupations in engineering and technology related fields have higher salaries than those in nursing, teaching and social work. (R)
7. We do NOT need to try to increase the pay of nurses, teachers and social workers to match those of engineers and computer scientists. (R)

### Support for Increasing Gender Balance

Men and women are currently unevenly distributed in different occupations. While there are more women in healthcare, teaching and social service professions, there are more men in engineering, technology and upper management professions. Please indicate the degree to which YOU agree or disagree with the following statements?

1. Occupations like nursing, teaching, and social work should be actively recruiting more men into such roles.
2. There should be more training programs in place to promote gender equality in fields where men are under-represented.
3. Policies should be enacted to encourage hiring more men in jobs where they are fewer in number, such as nursing, teaching, and social work.
4. Governments should commit resources toward changing the uneven gender distributions in fields like nursing, teaching, and social work.
5. Professions such as nursing, teaching, and social work would be enhanced with a more equal distribution of men and women.
6. Greater gender equality in currently female-dominated occupations would be beneficial to society as a whole.
7. Men would benefit if they were more equally represented in professions such as nursing, teaching and social work.
8. Women would benefit if men were more equally represented in professions such as nursing, teaching and social work.
9. Children would benefit if men were more equally represented in professions such as nursing, teaching and social work.
10. Those served by nurses, teachers or social workers would benefit if men were equally represented in such professions.
11. Occupations like engineering, computing, and management should be actively recruiting more women into such roles.
12. There should be more training programs in place to promote gender equality in fields where women are under-represented.
13. Policies should be enacted to encourage hiring more women in jobs where they are fewer in number, such as engineering, computing, and management.
14. Governments should commit resources toward changing the uneven gender distributions in fields like engineering, computing, and management.
15. Professions such as engineering, computing, and management would be enhanced with a more equal distribution of men and women.
16. Greater gender equality in male-dominated occupations would be beneficial to society as a whole.
17. Men would benefit if women were more equally represented in professions such as engineering, computing, and management.
18. Women would benefit if they were more equally represented in professions such as engineering, computing, and management.
19. Children would benefit if women were more equally represented in professions such as engineering, computing, and management.
20. Those served by engineers, computer specialists, and those in management positions would benefit if women were equally represented in such professions.
