# Supplementary material for: Worth Less?: Why Men (and Women) Devalue Care-Oriented Careers
Source: Front Psychol. 2018 Aug 10;9:1353. doi: 10.3389/fpsyg.2018.01353 (PMC6095964; doi:10.3389/fpsyg.2018.01353)
Supplement: Supplementary file 1 [file Table_1.docx]

Supplementary Online Materials

“Worth Less?: Why Men (and Women) Devalue Care-Oriented Careers”

Katharina Block

Toni Schmader

Alyssa Croft

*Table of Contents*

|  | **Study** | **Page** |
| --- | --- | --- |
| **List of Measures** |  |  |
|  | Study 1 | 3 |
|  | Study 2 | 3 |
|  | Study 3 | 3 |
| **Analyses of Condition Effects** |  |  |
|  | Study 2 | 5 |
| **Results without Control Variables** |  |  |
|  | Study 1 | 6 |
|  | Study 2 | 7 |
|  | Study 3 | 8 |
| **Results for STEM Perceptions** |  |  |
|  | Study 1 | 9 |
|  | Study 2 | 10 |
|  | Study 3 | 11 |
| **Results for Domestic Outcomes** |  |  |
|  | Study 1 | 12 |
|  | Study 2 | 13 |

**Full List of Measures in Order of Collection**

We list all variables measured in each study. Those with a * are analyzed or reported in the main manuscript. Data for this Project are available at osf.io/ejz78

**Study 1 Full List of Measures**

- Communal and agentic values*
- STEM and HEED interest*
- STEM and HEED worth to society*
- Anticipated family vs. career orientation
- Anticipated likelihood of becoming caregiver
- Anticipated likelihood of becoming breadwinner
- Demographics
  - Age
  - Gender*
  - Year in school
  - Sexual orientation
  - Ethnicity
  - Marital Status

**Study 2 Full List of Measures**

- Choice of Card to play in game
- Communal and agentic values*
- STEM and HEED interest*
- STEM and HEED worth to society*
- Anticipated family vs. career orientation
- Anticipated likelihood of becoming caregiver
- Anticipated likelihood of becoming breadwinner
- Trait competitiveness*
- Demographics
  - Age
  - Gender*
  - Year in school
  - Sexual orientation
  - Ethnicity
  - Major
  - Political Orientation
  - Marital Status

**Study 3 Full List of Measures**

- Communal, Agentic, & Material Values*
- Importance of Gross-Domestic Well-being (GDW) vs. Gross Domestic Product
- Expected relationship between GDP and GDW
- Actual hourly pay of HEED*, STEM*, Control Careers and Housework Roles
- Actual workhours of HEED*, STEM*, Control Careers and Housework Roles
- Worth to society of HEED*, STEM*, Control Careers and Housework Roles
- HEED*, STEM, * Control Careers and Housework Roles’ contribution to GDP vs. GDW
- Support for salary increases in HEED*
- Support for increased gender balance in HEED and STEM*
- Perceived compatibility of communal and agentic values
- Demographics:
  - Age
  - Gender*
  - Faculty
  - Year standing
  - Sexual orientation
  - Ethnicity
  - Born in North America?
  - Age at which English was learned
  - Marital Status
  - Political Orientation
  - Belief in God
  - SES
- Self-reported effort during the study
- Self-reported beliefs about goal of the study

**Analyses of Condition Effects**

Study 2 was initially designed as an experimental test of the effects of cooperative vs. competitive mindsets on career evaluations. The two following tables show that condition did not significantly affect any key variables, and also did not change results from main analyses.

*Table S1.* Study 2 Condition Differences

|  | Means Condition | | Tests of Condition Differences | |
| --- | --- | --- | --- | --- |
|  | Cooperate | Compete | t-value | p-value |
| Communal Values | 73.74 | 73.55 | 0.13 | .894 |
| Agentic Values | 66.76 | 69.42 | -1.68 | .094 |
| Competitiveness | 59.02 | 58.73 | 0.13 | .900 |
| HEED interest | 47.86 | 46.16 | 0.73 | .467 |
| HEED worth | 122.99 | 120.64 | 0.24 | .811 |
| STEM interest | 38.83 | 36.97 | 0.83 | .407 |
| STEM worth | 142.56 | 144.79 | -0.22 | .825 |

*Table S2. Study 2 HEED interest and HEED worth paths controlling for condition.*

| *Predictors* | *Outcome Variable* | | | | | | | | | | | | |
| --- | --- | --- | --- | --- | --- | --- | --- | --- | --- | --- | --- | --- | --- |
|  |  | Communal Values | | |  | HEED Interest | | |  | HEED worth | | | |
|  |  | *Beta* | *.95 CI* | *p-value* |  | *Beta* | *.95 CI* | *p-value* |  | *Beta* | *.95 CI* | *p-value* |  |
| Gender |  | **0.15** | **0.04 – 0.26** | **.010** |  | **0.43** | **0.33 – 0.52** | **<.001** |  | **0.07** | **0.01 – 0.14** | **.026** |  |
| Condition |  | -0.01 | -0.23 – 0.21 | .919 |  | -0.01 | -0.20 – 0.18 | .899 |  | -0.03 | -0.16 – 0.10 | .642 |  |
| Communal Values |  |  |  |  |  | **0.28** | **0.19 – 0.38** | **<.001** |  | **0.09** | **0.02 – 0.15** | **.010** |  |
| Agentic Values |  |  |  |  |  | **-0.17** | **-0.27 – -0.08** | **<.001** |  | **-0.08** | **-0.14 – -0.01** | **.022** |  |
| STEM Interest |  |  |  |  |  | **0.24** | **0.14 – 0.33** | **<.001** |  |  |  |  |  |
| STEM worth |  |  |  |  |  |  |  |  |  | **0.82** | **0.75 – 0.88** | **<.001** |  |
| Observations |  | 308 | | |  | 308 | | |  | 308 | | | |
| R^2^ / adj. R^2^ |  | .022 / .015 | | |  | .323 / .312 | | |  | .682 / .677 | | | |

**Results without Control Variables**

In our key analyses reported in the main manuscript, we control for perceptions of STEM careers in our relevant HEED outcome variables. By doing so, we assume we are controlling for individual differences in the extent to which participants tended to generally feel that careers are interesting and worthy of high pay. In addition, main analyses also enter communal values as our key predictor along with all other measured values in each study. For each study, the next three tables show key paths without any control variables. All conclusions remain the same.

| Predictors | Outcome Variable | | | | | | | | | | | | |
| --- | --- | --- | --- | --- | --- | --- | --- | --- | --- | --- | --- | --- | --- |
|  |  | Communal Values | | |  | HEED interest | | |  | HEED worth | | | |
|  |  | *Beta* | *.95 CI* | *p-value* |  | *Beta* | *.95 CI* | *p-value* |  | *Beta* | *.95 CI* | *p-value* |  |
| Gender |  | **0.23** | **0.13 – 0.33** | **<.001** |  | **0.28** | **0.18 – 0.37** | **<.001** |  | **0.11** | **0.01 – 0.22** | **.027** |  |
| Communal Values |  |  |  |  |  | **0.20** | **0.11 – 0.30** | **<.001** |  | **0.16** | **0.06 – 0.27** | **.002** |  |
| Observations |  | 379 | | |  | 379 | | |  | 379 | | | |
| R^2^ / adj. R^2^ |  | .053 / .051 | | |  | .145 / .141 | | |  | .049 / .044 | | | |

*Table S3. Study 1 paths for HEED interest and HEED worth without control variables*

*Table S4. Study 2 paths for HEED interest and HEED worth without control variables*

| *Predictors* | *Outcome Variable* | | | | | | | | | | | |
| --- | --- | --- | --- | --- | --- | --- | --- | --- | --- | --- | --- | --- |
|  |  | Communal Values | | |  | HEED interest | | |  | HEED worth | | |
|  |  | *Beta* | *.95 CI* | *p-value* |  | *Beta* | *.95 CI* | *p-value* |  | *Beta* | *.95 CI* | *p-value* |
| (Intercept) |  | 0.00 | -0.11 – 0.11 | 1.00 |  | -0.00 | -0.10 – 0.10 | 1.00 |  | -0.00 | -0.11 – 0.11 | 1.00 |
| Gender |  | **0.15** | **0.04 – 0.26** | **.009** |  | **0.39** | **0.29 – 0.49** | **<.001** |  | 0.08 | -0.03 – 0.20 | .150 |
| Communal Values |  |  |  |  |  | **0.25** | **0.15 – 0.35** | **<.001** |  | **0.12** | **0.00 – 0.23** | **.045** |
| Observations |  | 308 | | |  | 308 | | |  | 308 | | |
| R^2^ / adj. R^2^ |  | .022 / .019 | | |  | .242 / .237 | | |  | .023 / .017 | | |

**Study 3 Key Results without Controls**

*Table S5. Study 3 paths for HEED outcomes without control variables*

| *Predictors* | *Outcome Variable* | | | | | | | | | | | | | | | |
| --- | --- | --- | --- | --- | --- | --- | --- | --- | --- | --- | --- | --- | --- | --- | --- | --- |
|  |  | Communal Values | | |  | HEED worth | | |  | Salary Increase Support | | |  | Support for HEED Gender Balance | | |
|  |  | *Beta* | *.95 CI* | *p-value* |  | *Beta* | *.95 CI* | *p-value* |  | *Beta* | *.95 CI* | *p-value* |  | *Beta* | *.95 CI* | *p-value* |
| (Intercept) |  | -0.00 | -0.11 – 0.11 | 1.00 |  | -0.00 | -0.11 – 0.11 | 1.00 |  | 0.00 | -0.11 – 0.11 | 1.00 |  | -0.00 | -0.11 – 0.11 | 1.00 |
| Gender |  | **0.12** | **0.01 – 0.24** | **.033** |  | **0.21** | **0.09 – 0.32** | **<.001** |  | **0.33** | **0.22 – 0.43** | **<.001** |  | **0.35** | **0.25 – 0.46** | **<.001** |
| Communal Values |  |  |  |  |  | 0.07 | -0.05 – 0.18 | .246 |  | **0.21** | **0.11 – 0.32** | **<.001** |  | **0.19** | **0.08 – 0.29** | **<.001** |
| Observations |  | 291 | | |  | 291 | | |  | 291 | | |  | 291 | | |
| R^2^ / adj. R^2^ |  | .016 / .012 | | |  | .051 / .044 | | |  | .171 / .165 | | |  | .175 / .169 | | |

**Results for Perceptions of STEM Careers**

In all studies, participants rated their perceptions of STEM careers parallel to their perceptions of HEED careers. The following tables display regressions analyses parallel to the b-paths of the mediation analyses reported for HEED in the main manuscript. Entered as simultaneous predictors, regression analyses tested how gender, communal, values, and an additional value (depending on Study) predicted each outcome.

*Table S6. Study 1 paths for STEM interest and STEM worth*

| Predictors | Outcome Variable | | | | | | | |
| --- | --- | --- | --- | --- | --- | --- | --- | --- |
|  |  | STEM interest | | |  | STEM worth | | |
|  |  | *Beta* | *.95 CI* | *p-value* |  | *Beta* | *.95 CI* | *p-value* |
| (Intercept) |  | 0.00 | -0.09 – 0.10 | .948 |  | 0.00 | -0.07 – 0.07 | .992 |
| Gender |  | **-0.26** | **-0.36 – -0.16** | **<.001** |  | -0.02 | -0.09 – 0.05 | .519 |
| Communal Values |  | **-0.12** | **-0.22 – -0.02** | **.017** |  | -0.05 | -0.12 – 0.02 | .150 |
| Agentic Values |  | 0.02 | -0.07 – 0.11 | .671 |  | **0.09** | **0.02 – 0.15** | **.010** |
| HEED Interest |  | **0.39** | **0.29 – 0.49** | **<.001** |  |  |  |  |
| HEED Worth |  |  |  |  |  | **0.77** | **0.70 – 0.84** | **<.001** |
| Observations |  | 378 | | |  | 378 | | |
| R^2^ / adj. R^2^ |  | .156 / .147 | | |  | .588 / .584 | | |

*Table S7. Study 2 paths for STEM interest and STEM worth*

| Predictors | Outcome Variable | | | | | | | |
| --- | --- | --- | --- | --- | --- | --- | --- | --- |
|  |  | STEM interest | | |  | STEM worth | | |
|  |  | *Beta* | *.95 CI* | *p-value* |  | *Beta* | *.95 CI* | *p-value* |
| (Intercept) |  | 0.00 | -0.11 – 0.11 | 1.00 |  | 0.00 | -0.06 – 0.06 | 1.00 |
| Gender |  | **-0.30** | **-0.42 – -0.17** | **<.001** |  | -0.05 | -0.12 – 0.02 | .138 |
| Communal Values |  | **-0.13** | **-0.24 – -0.01** | **.032** |  | **-0.06** | **-0.13 – 0.01** | **.073** |
| Agentic Values |  | **0.12** | **0.00 – 0.23** | **.044** |  | **0.10** | **0.03 – 0.17** | **.005** |
| Trait Competitiveness |  | -0.05 | -0.17 – 0.08 | .462 |  | 0.01 | -0.06 – 0.08 | .795 |
| HEED interest |  | **0.32** | **0.19 – 0.44** | **<.001** |  |  |  |  |
| HEED worth |  |  |  |  |  | **0.82** | **0.76 – 0.89** | **<.001** |
| Observations |  | 308 | | |  | 308 | | |
| R^2^ / adj. R^2^ |  | .104 / .089 | | |  | .679 / .674 | | |

*Table S7. Study 3 paths for STEM worth and Support for Gender Balance*

| Predictors | Outcome Variable | | | | | | | |
| --- | --- | --- | --- | --- | --- | --- | --- | --- |
|  |  | STEM Worth | | |  | STEM Gender Balance Support | | |
|  |  | *Beta* | *.95 CI* | *p-value* |  | *Beta* | *.95 CI* | *p-value* |
| (Intercept) |  | 0.00 | -0.06 – 0.06 | 1.00 |  | 0.00 | -0.08 – 0.08 | 1.00 |
| Gender |  | -0.03 | -0.09 – 0.04 | .405 |  | **0.21** | **0.12 – 0.30** | **<.001** |
| Communal Values |  | -0.05 | -0.11 – 0.02 | .157 |  | 0.06 | -0.03 – 0.14 | .205 |
| Agentic Values |  | 0.02 | -0.06 – 0.11 | .574 |  | -0.03 | -0.15 – 0.08 | .551 |
| Material Values |  | **0.10** | **0.02 – 0.19** | **.017** |  | -0.02 | -0.13 – 0.10 | .783 |
| Perceived Salary |  | **0.47** | **0.39 – 0.54** | **<.001** |  | 0.02 | -0.07 – 0.11 | .635 |
| Perceived Workhours |  | **0.19** | **0.12 – 0.25** | **<.001** |  | -0.05 | -0.14 – 0.04 | .256 |
| HEED worth |  | **0.40** | **0.32 – 0.48** | **<.001** |  |  |  |  |
| HEED Gender Equality Support |  |  |  |  |  | **0.59** | **0.50 – 0.68** | **< .001** |
| Observations |  | 291 | | |  | 291 | | |
| R^2^ / adj. R^2^ |  | .745 / .738 | | |  | .527 / .515 | | |

**Results for Domestic Outcomes**

In Study 1 and Study 2, we also collected exploratory measures that assessed the extent to which participants expected to fulfill care-oriented communal roles in their future families. The following three tables show how these variables were predicted by gender and communal values independently.

Table S8. *Study 1 Predicting Domestic Outcomes.*

| Predictors | Outcome Variable | | | | | | | | | | | | |
| --- | --- | --- | --- | --- | --- | --- | --- | --- | --- | --- | --- | --- | --- |
|  |  | Family-Orientation | | |  | Likelihood Caregiver | | |  | Likelihood Breadwinner | | | |
|  |  | *Beta* | *.95 CI* | *p-value* |  | *Beta* | *.95 CI* | *p-value* |  | *Beta* | *.95 CI* | | *p-value* |
| (Intercept) |  | -0.00 | -0.10 – 0.09 | .960 |  | -0.01 | -0.11 – 0.09 | .867 |  | -0.01 | -0.11 – 0.08 | | .800 |
| Gender |  | 0.02 | -0.08 – 0.12 | .723 |  | 0.25 | 0.15 – 0.35 | <.001 |  | **-0.43** | **-0.53 – -0.34** | **<.001** | |
| Communal Values |  | **0.33** | **0.43 – 0.23** | **<.001** |  | **0.13** | **0.02 – 0.23** | **.022** |  | **0.13** | **0.03 – 0.23** | | **.014** |
| Agentic Values |  | **0.21** | **0.12 – 0.31** | **<.001** |  | 0.06 | -0.04 – 0.16 | .204 |  | 0.07 | -0.02 – 0.16 | | .145 |
| Observations |  | 378 | | |  | 354 | | |  | 362 | | | |
| R^2^ / adj. R^2^ |  | .129 / .122 | | |  | .099 / .091 | | |  | .189 / .182 | | | |

Table S9. *Study 2 Predicting Domestic Outcomes.*

| Predictors | Outcome Variable | | | | | | | | | | | |
| --- | --- | --- | --- | --- | --- | --- | --- | --- | --- | --- | --- | --- |
|  |  | Family-Orientation | | |  | Likelihood Caregiver | | |  | Likelihood Breadwinner | | |
|  |  | *Beta* | *.95 CI* | *p-value* |  | *Beta* | *.95 CI* | *p-value* |  | *Beta* | *.95 CI* | *p-value* |
| (Intercept) |  | -0.00 | -0.11 – 0.11 | 1.00 |  | -0.00 | -0.10 – 0.10 | 1.00 |  | 0.00 | -0.09 – 0.09 | 1.00 |
| Gender |  | 0.10 | -0.01 – 0.21 | .076 |  | **0.43** | **0.33 – 0.53** | **<.001** |  | **-0.57** | **-0.66 – -0.47** | **<.001** |
| Communal Values |  | **0.20** | **0.09 – 0.31** | **<.001** |  | 0.08 | -0.02 – 0.18 | .130 |  | -0.00 | -0.10 – 0.09 | .932 |
| Agentic Values |  | **-0.23** | **-0.33 – -0.12** | **<.001** |  | 0.07 | -0.03 – 0.17 | .184 |  | **0.11** | **0.01 – 0.20** | **.026** |
| Observations |  | 308 | | |  | 308 | | |  | 308 | | |
| R^2^ / adj. R^2^ |  | .093 / .084 | | |  | .213 / .205 | | |  | .327 / .320 | | |
